# Substrate and inhibitor specificity of *Plasmodium* nucleoside transporters ENT1 orthologs

**DOI:** 10.1016/j.jbc.2024.108115

**Published:** 2024-12-24

**Authors:** Xinyi Chen, Chengyu Tian, Yingying He, Yaozong Li, Yanxia Zhou, Xiang Wang, Mi Zhou, Jingwen Lin, Zhong Lian, Dong Deng

**Affiliations:** 1 Department of Obstetrics, Key Laboratory of Birth Defects and Related Disease of Women and Children of MOE, State Key Laboratory of Biotherapy, West China Second Hospital, Sichuan University, Chengdu, China; 2 West China Biomedical Big Data Center, West China Hospital, Sichuan University, Chengdu, China; 3 Department of Chemistry, Umeå University, Umea, Sweden; 4 Department of Biochemistry, University of Zurich, Zurich, Switzerland; 5 West China School of Basic Medical Sciences & Forensic Medicine, Sichuan University, Chengdu, China; 6 State Key Laboratory of Biotherapy and Cancer Center, West China Hospital, Sichuan University, Chengdu, China; 7 NHC Key Laboratory of Chronobiology, Sichuan University, Chengdu, China

## Abstract

Malaria caused by *Plasmodium* infection poses a serious hazard to human health. *Plasmodium falciparum* equilibrative nucleoside transporter 1 (PfENT1), which mediates nucleoside uptake, is essential for the growth and proliferation of *Plasmodium* parasites, suggesting that PfENT1 is a potential antimalarial target. The promising compound GSK4 effectively inhibits the transport activity of PfENT1, thereby restraining the growth of *Plasmodium* parasites. However, it still needs to be clarified whether *Plasmodium* ENT1 orthologs have different selectivities for nucleosides and inhibitors. Here, we systematically compared the nucleoside selectivity of *Plasmodium* ENT1 orthologs from *P. falciparum* (PfENT1), *Plasmodium berghei* (PbENT1)*,* and *Plasmodium vivax* (PvENT1), revealing that *Plasmodium* ENT1 orthologs present a distinct nucleoside recognition pattern. In addition, GSK4 robustly binds to PfENT1 and PvENT1 from two human-hosted *Plasmodium* parasites but has a weakened binding affinity for PbENT1 from mouse-hosted *Plasmodium* parasites. We further structurally optimized the inhibitor and generated three GSK4 analogs. One of the GSK4 analogs presented a slightly increased binding affinity for PfENT1. This optimization represents a promising advancement for antimalarial drug development, providing a novel foundation for future endeavors in antimalarial drug design.

Malaria is an infectious disease caused by *Plasmodium* infection (1, 2). In 2022, approximately 2.49 billion people were at risk for malaria infection, and 600,000 people died from malaria (3). Two species, *Plasmodium falciparum* and *Plasmodium vivax*, cause the majority of cases of human malaria (4, 5). Moreover, *Plasmodium berghei* is a mouse-hosted malaria parasite that is a great model and plays a vital role in human malaria research (6).

Purines play an essential role in cellular metabolic processes, including the synthesis of ATP, RNA, and DNA (7, 8). Nevertheless, *Plasmodium* parasites are purine auxotrophs incapable of *de novo* purine biosynthesis (9, 10, 11). Therefore, the parasites salvage purines from the host cells *via* the nucleoside transporters. Up to date, there are two kinds of nucleoside transporters identified in eukaryotes, including the concentrative nucleoside transporter (sodium-coupled transporters) and the equilibrative nucleoside transporter (ENT, nucleoside facilitator) (12, 13). However, only four ENT homologs (ENT1-4) were identified in *P. falciparum* (14). PfENT1, located predominantly in the parasite plasma membrane, is the primary nucleoside import pathway for *P. falciparum* (11, 15, 16, 17, 18, 19, 20, 21, 22, 23, 24). Several leading compounds have been shown to inhibit the purine uptake of PfENT1 and suppress the proliferation of malaria parasites. Hence, PfENT1 is a potential target for novel antimalarial drugs.

In our previous work, we reported the cryo-EM structures of PfENT1 in the apo, inosine-bound, and inhibitor-bound states at resolutions of 3.3 Å, 3.1 Å, and 4 Å, respectively (25). The captured PfENT1 with eleven transmembrane helixes presents an inward-open conformation. The nucleoside- and inhibitor-binding sites partially overlap in the cavity between the N-terminal and C-terminal domains. By combining systematic biochemical and biophysical analyses, we revealed the nucleoside recognition mechanism of PfENT1 and the inhibition mechanism of GSK4 (25).

PfENT1 shares 75% sequence identity with *P. vivax* ENT1 (PvENT1) and 60% identity with *P. berghei* ENT1 (PbENT1) (Fig. S1). The recently described inhibitors of PfENT1 also exhibit affinities in targeting PvENT1 and PbENT1 (26, 27, 28). It needs to be clarified whether GSK4, a well-defined PfENT1 inhibitor, has a similar binding affinity to PvENT1 and PbENT1 (29). Additionally, the nucleoside selectivities of PvENT1 and PbENT1 remain unknown. Understanding how different ENT1 orthologs recognize and import their substrates could contribute to a deeper comprehension of nucleoside transport mechanisms and inform the design of antimalarial drugs.

## Results

### Characterization of *Plasmodium* ENT1 orthologs

**Figure 1:**
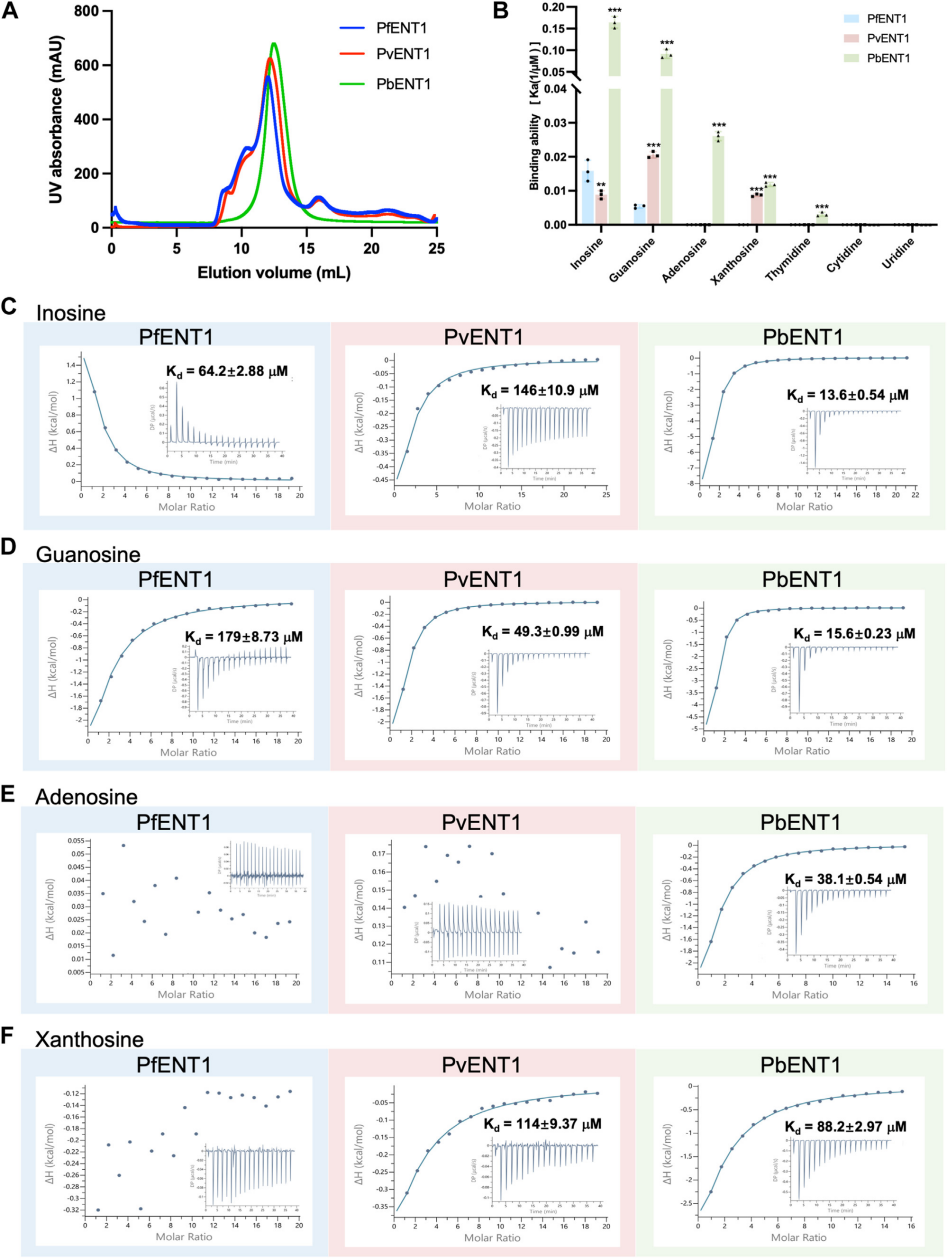
**Characterization of ENT1 orthologs.***A*, purification of PfENT1, PvENT1, and PbENT1 *via* size-exclusion chromatography (Superdex 200 10/300 Increase). *B*, the quantified results of ITC-binding data. ∗*p* < 0.05, ∗∗*p* < 0.01, and ∗∗∗*p* < 0.001 *versus* the PfENT1 group. *C*, inosine binding of ENT1 orthologs measured by ITC. *D*, guanosine binding of ENT1 orthologs measured by ITC. *E*, adenosine binding of ENT1 orthologs measured by ITC. *F*, Xanthosine binding of ENT1 orthologs measured by ITC. The error bars were obtained from a fit of the data points of the particular ITC experiments.

Recently, we purified PfENT1 from Sf9 insect cells (25). Therefore, we purified the other two orthologs, PvENT1 and PbENT1, *via* a similar strategy. As a result, we obtained purified PvENT1 and PbENT1 with monodisperse peaks in the detergent micelles (Fig. 1*A*, Fig. S2). Notably, three proteins were eluted at an elution volume of approximately 12 ml, suggesting that they share an identical oligomeric state in the detergent micelles (Fig. 1*A*, Fig. S2).

We conducted isothermal titration calorimetry (ITC) to measure the binding affinities of *Plasmodium* ENT1 orthologs with various nucleosides, including inosine, guanosine, adenosine, xanthosine, thymidine, cytidine, and uridine (Fig. 1*B*, Fig. S3, Table S1). As a result, we determined the binding affinities of PfENT1 for inosine and guanosine to be approximately 64.2 μM and 179 μM, respectively. The binding between PfENT1 and other nucleosides was undetectable (25). Notably, compared with PfENT1, PvENT1 exhibited a greater binding affinity for guanosine (approximately 49.3 μM) but a lower binding affinity for inosine (approximately 146 μM) (Fig. 1, *C* and *D*). Of note, the binding between PvENT1 and xanthosine is 114 μM. Consistently, the binding affinities between PvENT1 and pyrimidine nucleosides were undetectable (Fig. 1*E*, Fig. S3, Table S1). Interestingly, PbENT1 binds to inosine and guanosine with similar binding affinities of 13.6 μM and 15.6 μM, respectively. Therefore, compared with PfENT1 and PvENT1, PbENT1 has a stronger binding affinity for inosine and guanosine (Fig. 1, *C* and *D*). Moreover, the binding affinity of PbENT1 for adenosine and xanthosine are 38.1 μM and 88.2 μM, respectively. In contrast, its binding affinity to thymidine is weak (Fig. S3). No binding affinity was detected for cytidine or uridine (Fig. S3). Furthermore, a competition assay revealed that adenosine and thymidine effectively inhibited the inosine transport activity of PfENT1 despite undetectable binding affinities between adenosine/thymidine and PfENT1 (25), which suggests that these nucleosides may compete for the inosine-binding site of PfENT1. In contrast, PbENT1 demonstrated binding affinities with adenosine and thymidine, indicating its ability to bind multiple nucleosides efficiently. Our systematic binding assays suggested that PvENT1 prefers guanosine rather than inosine, whereas PbENT1 exhibits broader nucleoside selectivity with higher affinities for both inosine and guanosine. These findings indicate the specific nucleoside recognition preferences exhibited by *Plasmodium* ENT1 orthologs.

### Structural basis for the specific recognition of nucleosides by ENT1 orthologs

**Figure 2:**
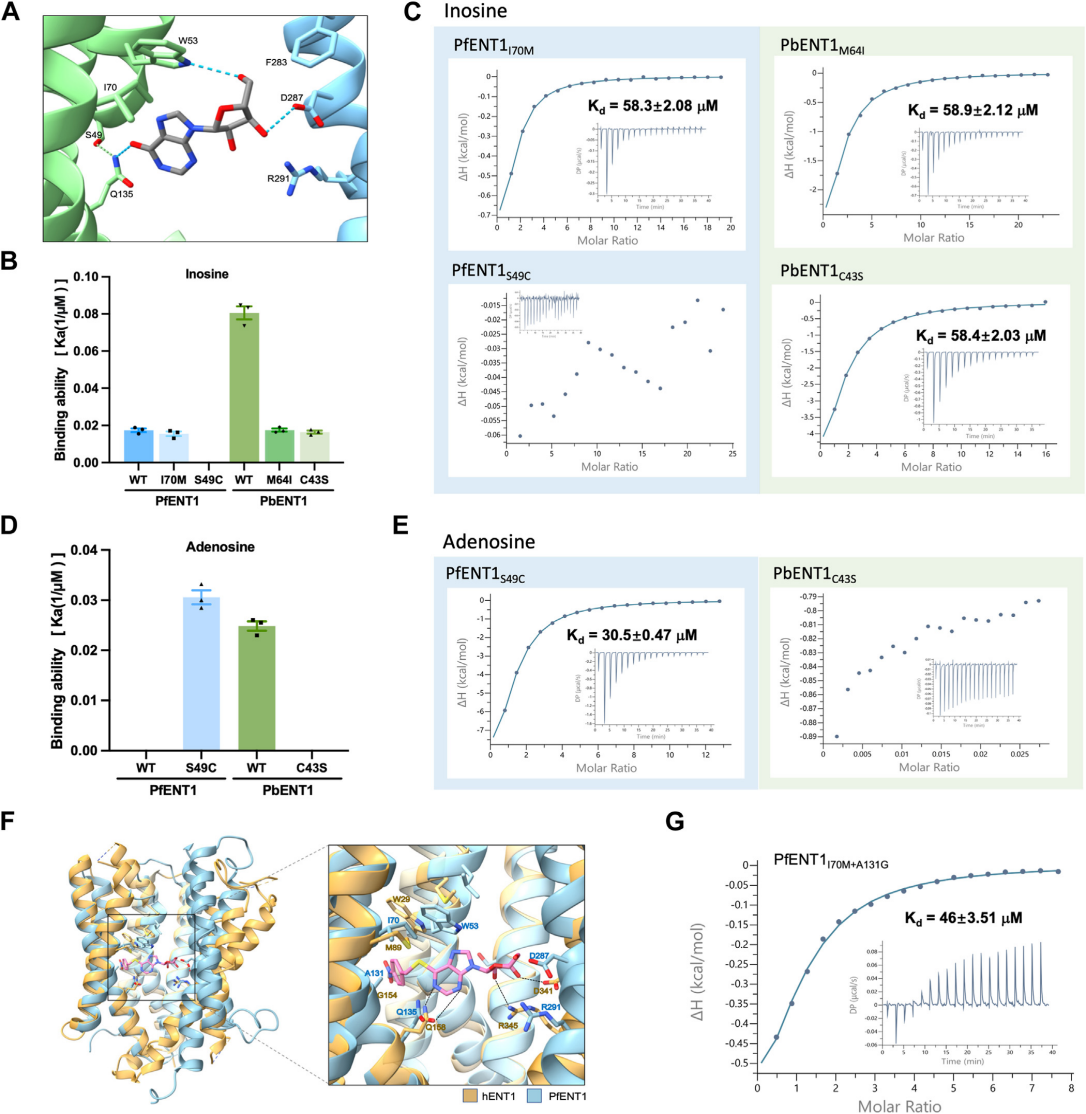
**Structural basis for nucleoside-specific recognition.***A*, inosine-binding site of PfENT1. The *dashed* lines denote the inosine coordinates in PfENT1. *B*, the quantified results of inosine-binding data. *C*, inosine binding of PfENT1_I70M/S49C_ and PbENT1_M64I/C43S_ measured by ITC. *D*, quantification of the adenosine-binding data. *E*, adenosine binding of PfENT1_S49C_ and PbENT1_C43S_ measured by ITC. *F*, superposition of the NBMPR-bound hENT1 complex and PfENT1. The *dashed* lines denote the NBMPR coordinates in hENT1. *G*, adenosine binding of PfENT1_I70M + A131G_ measured by ITC.

Structural information on PfENT1 in complex with inosine provides an opportunity to analyze and explore the differences among the three ENT1 orthologs (25) (Fig. 2*A*). Interestingly, we found that those of PvENT1 are identical to the substrate recognition residues of PfENT1. However, the hydrophobic residue Ile70 of PfENT1 is substituted into methionine (Met64) in PbENT1, and the vital residue Ser49 of PfENT1, which interacts with Gln135 to stabilize the binding of inosine, is substituted into cysteine (Cys43) in PbENT1 (Fig. S1).

To ascertain the crucial role of residues Ile70 and Ser49 in nucleoside binding, we introduced substitutions in PfENT1 and PbENT1: PfENT1_I70M_, PfENT1_S49C_, PbENT1_M64I_, and PbENT1_C43S_. Notably, PfENT1_I70M_ retained a comparable affinity for inosine with endothermic and exothermic action transitions during inosine titration. Moreover, PbENT1_M64I_ binds to inosine with a binding affinity of 58.9 μM, fivefold lower than WT PbENT1. Furthermore, the results revealed complete abolishment of the inosine binding of PfENT1_S49C._ Furthermore, PbENT1_C43S_ binds to inosine with a binding affinity of 58.4 μM, fivefold lower than WT PbENT1 (Fig. 2, *B* and *C*). Therefore, the residues Ile70 and Ser49 surrounding the substrate-binding pocket play a vital role in the inosine recognition of ENT1 orthologs.

Surprisingly, while adenosine binding was undetectable with PfENT1_WT_, PfENT1_S49C_ exhibited a binding affinity for adenosine of approximately 30.5 μM. In contrast, PbENT1_C43S_ did not show detectable adenosine binding, indicating a loss of binding ability. Mechanistically, the cysteine loses the ability to stabilize the Gln135, resulting in a dynamic and flipping side chain of Gln135. These findings suggest that Cys43 in PbENT1 is a critical residue for the recognition and binding of adenosine (Fig. 2, *D* and *E*).

In previous studies, human equilibrative nucleoside transporter 1 (hENT1) was shown to play a crucial role in adenosine transport (30). The two distinct residues (Met89 and Gly154) of hENT1 may contribute to the recognition of adenosine. Notably, the residues Ile70 and Ala131 of PfENT1 correspond to Met89 and Gly154 of hENT1, respectively (Fig. 2*F*). We further generated PfENT1_I70M/A131G_, which exhibited a binding affinity for adenosine of approximately 46 μM (Fig. 2*F*).

Interestingly, PfENT1_I70M/A131G_ and PfENT1_S49C_ demonstrated a similar binding capacity to adenosine. However, the thermodynamic parameters of adenosine titrations of two PfENT1 variants (PfENT1_I70M/A131G_ and PfENT1_S49C_) present apparent differences. The enthalpy-driven binding of PfENT1_S49C_ indicates that the specific noncovalent interaction between PfENT1_S49C_ and adenosine (31, 32), especially the hydrogen bond between PfENT1_S49C_ and adenosine, plays a significant role (Fig. S7*C*). The entropy-driven binding of PfENT1_I70M/A131G_ indicates an increase in the system's disorder, especially the hydrophobic interactions between PfENT1_I70M/A131G_ and adenosine. Notably, substitution A131G has potential changes to the structure of PfENT1 due to the appearance of two consecutive glycines (Fig. S1). The successive occurrence of two glycines causes the break of the transmembrane helix (without the hydrogen bond provided by the side chain), which in turn causes a subtle change in the direction of the transmembrane helix (33). Consequently, we identified several vital residues affecting the substrate binding and specificity of ENT1 orthologs.

### Inhibitor-specific recognition of ENT1 orthologs

The promising inhibitor GSK4 occupies the substrate-binding site of PfENT1 and extends the hydrophobic tail to block potential conformational changes during the alternating access cycle of PfENT1 (25). However, whether GSK4 can also bind to other ENT1 orthologs remains unclear.

**Figure 3:**
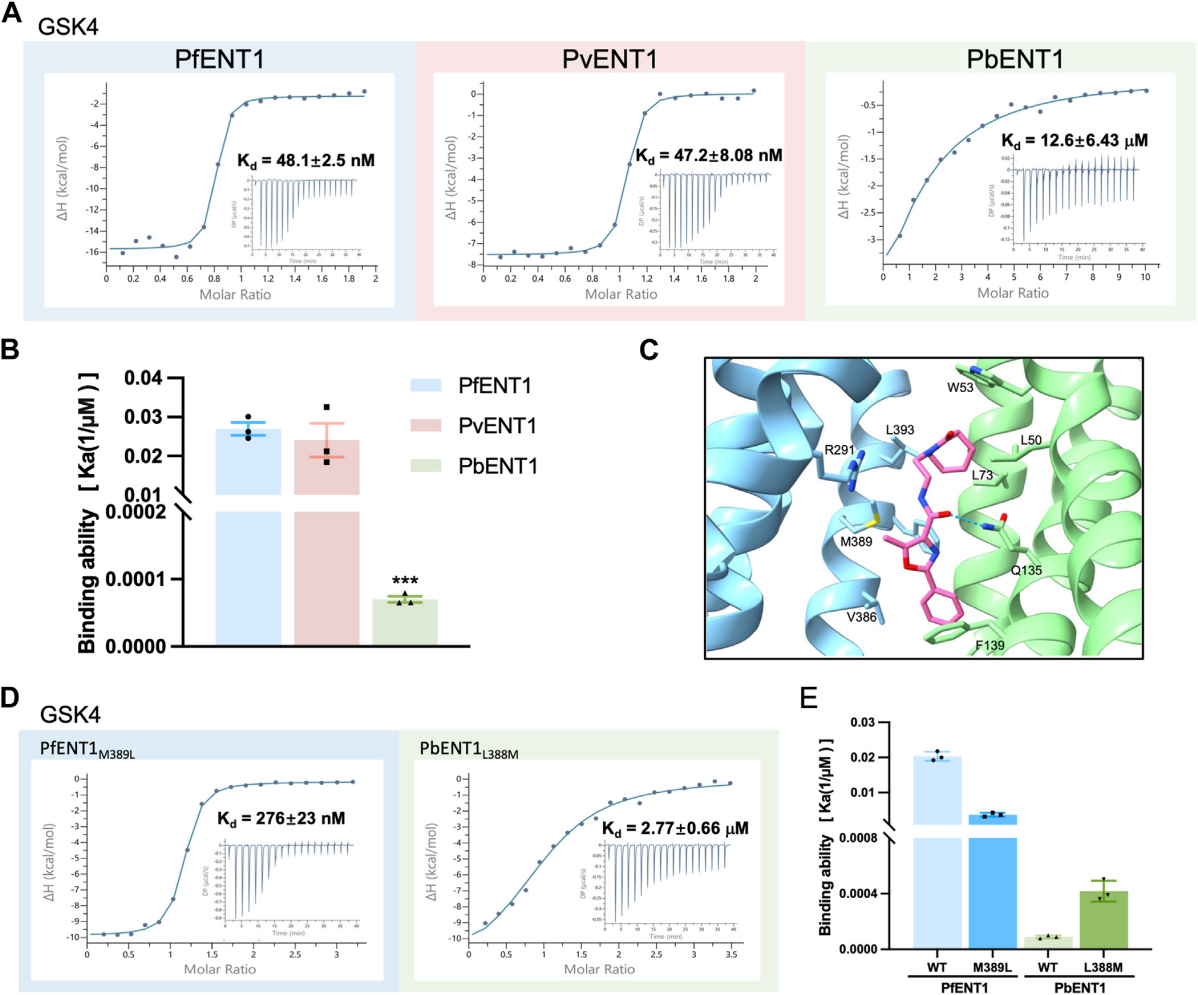
**Inhibitor-specific recognition of ENT1 orthologs.***A*, GSK4 binding of ENT1 orthologs measured by ITC. *B*, the quantified results of the GSK4-binding data. *C*, GSK4-binding site of PfENT1. The *dashed lines* denote GSK4 coordination in PfENT1. *D*, GSK4 binding of PfENT1_M389L_ and PbENT1_L388M_ measured by ITC. *E*, the quantified results of the GSK4-binding data from D.

To further validate the potential inhibitory activity of *Plasmodium* ENT1 orthologs, we measured the binding affinities between *Plasmodium* ENT1 orthologs and GSK4 *via* ITC. Consistent with previous studies, the binding affinity between PfENT1 and GSK4 was 48.1 nM (25). PvENT1 binds to GSK4 with a binding affinity of approximately 47.2 nM, comparable to PfENT1. However, PbENT1 has a significantly lower binding affinity for GSK4, with a value of 12.6 μM, which is markedly less than that of PfENT1 and PvENT1 and is comparable to the binding affinities of inosine or guanosine (Fig. 3, *A* and *B*). These findings suggest that GSK4 can inhibit multiple ENT1 orthologs.

To decipher the diversity of ENT1 orthologs that GSK4 can recognize, we compared the key residues of PfENT1, PvENT1, and PbENT1 (25) (Fig. 3*C*). Interestingly, there is no difference between PfENT1 and PvENT1. However, the Met389 residue of PfENT1 is replaced with Leu in PbENT1 at the corresponding position. To determine whether residue Met389 of PfENT1 plays a vital role in GSK4 binding, we generated PfENT1_M389L_ and measured its binding affinity with GSK4. Consequently, the binding affinity of PfENT1_M389L_ for GSK4 was 276 nM, which is approximately five times weaker than that of PfENT1_WT_. Additionally, the binding affinity of PbENT1_L388M_ for GSK4 was 2.77 μM, which was five times greater than that of PbENT1_WT_ (Fig. 3, *D* and *E*). These findings suggest that the differences in key residues between PfENT1 and PbENT1 are essential for the ability of ENT1 orthologs to bind to the inhibitor.

### Optimization of GSK4 analogs

**Figure 4:**
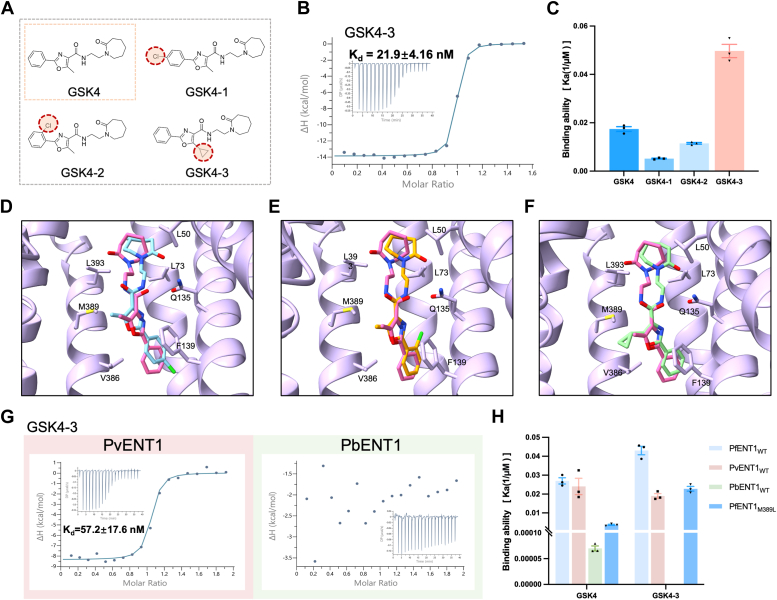
**Inhibitor optimization based on GSK4.***A*, structures of GSK4, GSK4-1, GSK4-2, and GSK4-3. *B*, binding of PfENT1 to GSK4-3 measured by ITC. *C*, quantification of GSK4 and GSK4 analog binding data. *D*, superposition of the GSK4-bound PfENT1 complex with GSK4-1, GSK4-2 (*E*), and GSK4-3 (*F*). *G*, binding of PvENT1 and PbENT1 to GSK4-3 measured by ITC. *H*, quantification of the GSK4 and GSK4-3 binding data.

The reported structure of PfENT1 in complex with GSK4 revealed an additional space adjacent to the central cavity of PfENT1 (Fig. 4, *D–F*) (25). Upon evaluating the chemical accessibility of GSK4, we chose to bolster the compound by introducing small substituents at the benzene and oxazole rings, including chlorine and cyclopropyl groups. Consequently, we synthesized the inhibitors GSK4-1, GSK4-2, and GSK4-3 (Fig. 4*A*, Fig. S6).

To validate the potency of these small molecules, we employed ITC to determine their binding parameters. GSK4-1 and GSK4-2 exhibited reduced binding affinities for PfENT1, with 3.5-fold and 1.6-fold decreases, respectively (Fig. 4*C*, Fig. S5, *A* and *B*). Notably, GSK4-3 demonstrated a remarkable 2.7-fold increase in binding affinity (Fig. 4, *B* and *C*). The introduced cyclopropyl moiety engages more effectively with surrounding residues through van der Waals interactions, notably Met389 and Phe362, elucidating the enhanced binding affinity. Crucially, this GSK4 analog also exhibited comparable binding to PvENT1 and GSK4 but negligible binding to murine PbENT1 (Fig. 4*G*). Furthermore, compared with its parent compound GSK4, the binding affinity of PfENT1_M389L_ for GSK4-3 was 44.4 nM, resulting in a 6-fold increase in binding affinity (Fig. 3*D*, Fig. S5*C* and Fig.4*H*). Notably, GSK4-3 has lower cytotoxicity than GSK4 and the other two GSK4 analogs (Fig. S8). Meanwhile, GSK4-3 cannot bind to the bacterial nucleoside transporter NupG (Fig. S8).

The dual inhibition ability and enhanced resistance of GSK4-3 indicate a promising direction for further optimization. Analysis of the binding parameters from detailed ITC data revealed that these modifications predominantly increase the entropic terms while minorly decreasing the enthalpic terms.

## Discussion

This study elucidated the distinct nucleoside recognition patterns of *Plasmodium* ENT1 orthologs. PfENT1 and PvENT1 exhibit high selectivity for inosine and guanosine, whereas PbENT1 shows broader specificity, binding multiple nucleosides with higher affinity. These findings increase our understanding of substrate recognition and provide a foundation for targeted antimalarial drug design. Although the binding preferences of PfENT1, the inosine transport of PfENT1 is inhibited by other nucleosides with undetectable binding (25). A similar phenomenon was observed in the investigation of human ENT1 (34).

In this study, we conducted systematic ITC experiments, which provided us with Gibbs free energy, enthalpy change, and entropy change of substrate or inhibitor binding. PfENT1 interacts with inosine, presenting an endothermic reaction (ΔH>0). However, PvENT1/PbENT1 interacts with inosine, presenting an exothermic reaction (ΔH<0) (Fig. S7*A*). This suggests that the three ENTs and inosine interactions are three different patterns, including the entropy-driven (PfENT1-inosine), the enthalpy-entropy compensation (PvENT1-inosine), and the enthalpy-driven (PbENT1-inosine). During the binding process, the ligands remove the solvated water molecules, or the degrees of freedom of the system increase, which will lead to an increase in entropy. However, the structure of the PfENT1–inosine complex has only been obtained at a low resolution. Structural information on PvENT1 and PbENT1 was also lacking—these limit the determination of the water-mediated interactions in the ENT1 orthologs–nucleosides complex. In addition, the possible conformations of ENT1 in detergent micelle may affect the enthalpy and entropy changes of nucleoside and transporter interactions. Therefore, the reasons for the three different driving modes need to be further studied.

Previous studies have shed light on the potential of GSK4 as an inhibitor of PfENT1 for treating malaria and offering insights into developing antimalarial drugs (25). Our investigations also demonstrated that GSK4 effectively binds to PvENT1 with a binding affinity at 47.2 nM, suggesting GSK4 inhibits the nucleoside transport mediated by PvENT1, and GSK4 is a potential antimalarial compound against two major human-hosted *Plasmodium* parasites. Notably, there are potential differences in inhibitor-binding ability between *Plasmodium* species that infect humans and those that infect other hosts. The optimized GSK4 inhibitor has increased binding ability, suggesting a new avenue for developing antimalarial drugs. This optimization deepens our understanding of molecular interactions and provides a foundation for future drug design and refinement.

## Experimental procedures

### Protein expression and purification

All the constructs were subcloned and inserted into a modified pFastBac with an N-terminal 10 × His tag. The baculovirus was generated in DH10Bac for recombinant protein expression. After 72 h of infection, Sf9 insect cells were harvested. For structural determination of the PfENT1 protein, Sf9 cells were solubilized in buffer containing 25 mM MES (pH 6.0), 150 mM NaCl, 2% (w/v) n-dodecyl-β-D-maltopyranoside (DDM), and protease inhibitor cocktail (0.8 μM aprotinin, 2 μM pepstatin, and 5 μg/ml leupeptin) at 4 °C for 2 h. After centrifugation at 18,000 rpm for 45 min at 4 °C, the detergent-soluble supernatant was collected *via* centrifugation at 4 °C for 60 min and incubated with Ni-NTA resin (QIAGEN) at 4 °C for 20 min. The resin was further washed with 25 mM MES pH 6.0, 150 mM NaCl, 10 mM imidazole, and 0.05% (w/v) DDM and eluted with buffer containing 25 mM MES pH 6.0, 150 mM NaCl, 300 mM imidazole, and 0.05% (w/v) DDM. The protein was further purified *via* size-exclusion chromatography (Superdex 200 Increase 10/300; GE Healthcare) in buffer containing 25 mM MES (pH 6.0), 150 mM NaCl, and 0.05% (w/v) DDM. The peak fractions were pooled and concentrated to 10∼50 μM for the nucleoside-binding assay. The protein purification of the bacterial nucleoside transporter NupG was performed as described in previous studies (35).

### ITC experiments

Nucleotide binding to ENTs was measured at 25 °C *via* a MicroCal PEAQ-ITC. The purified ENT protein was concentrated to 25 to 50 μM for ITC experiments. The nucleoside was diluted to 5 mM *via* the same buffer employed for protein purification, which consisted of 25 mM MES (pH 6.0), 150 mM NaCl, and 0.05% DDM. The inhibitor was diluted to 250 μM *via* the same buffer employed for protein purification, which consisted of 25 mM MES (pH 6.0), 150 mM NaCl, and 0.05% DDM. All the titrations were repeated three times.

### In silico optimization

We chose a specific structure from the PDB (PDB: 7YDQ) as the basis for the compound optimization. This structure shows the binding mode of GSK4 to PfENT1. Owing to its relatively low resolution (4.04 Å), we decided to refine the binding mode locally. To this end, we first prepared the GSK4/PfENT1 complex by Maestro (version 11.5), including assigning bond orders and adding hydrogens. All the titratable residues were then protonated to their correct state at pH = 7.4 *via* ProPka-3.0 (36). The orientations of the polar residues of PfENT1 were fine-tuned manually according to detailed intermolecular and intramolecular contacts. To further refine the interactions between the ligand and protein, we redocked GSK4 to the prepared PfENT1. Before redocking, we drew the 2D structure of GSK4 with MarvinSketch (MarvinSketch 19.25, 2019, ChemAxon (http://www.chemaxon.com)) and generated its 3D coordinates in Maestro. The new poses were obtained *via* the docking software AutoDock Vina 1.1.2 (37). The docking site was defined by a 30 Å × 30 Å × 30 Å grid box and centered on the coordinates of GSK4. We set the parameter “exhaustiveness” to 50 to enhance the configurational sampling of binding poses and left the rest of the parameters as defaults. Finally, the 10 best-ranked poses were saved for further analysis. We added all hydrogens to the regenerated binding poses in Meastro and compared them with the native binding pose. We finally select the most reasonable pose as the starting point for the following optimization.

### Inhibitor synthesis procedure

A general experimental procedure for the synthesis of oxazoles *via* copper-catalyzed tandem oxidative cyclization: To a solution of benzylamine (2.0 equiv) derivatives in DMF, iodine (1.2 equiv), 1,3-dicarbonyl compounds (1.0 equiv), Cu(OAc)2·(0.2 equiv), and TBHP (2.0 equiv) were added. The reaction mixture was stirred for 4 h at room temperature. Upon completion, the reaction mixture was extracted with EtOAc and dried with Na_2_SO_4_. Then, the organic phase was concentrated under vacuum and purified by silica gel column chromatography to obtain the desired compound 1.

A solution of 1 (1.0 mmol) and sodium hydroxide (1.5 mmol) in ethanol was stirred at room temperature overnight. The ethanol was evaporated, and the residue was acidified (approximately pH 5) with 2 M hydrochloric acid and extracted with ethyl acetate. The combined organics were washed with brine, dried (MgSO_4_), evaporated, resulting in the generation of compound 2.

A two-neck round-bottom flask equipped with a mechanical stirrer and a dropping funnel was used. To a stirred solution of aryl acid in CH_2_Cl_2_ at room temperature, oxalyl chloride (2.0 equiv) and two drops of DMF were added, and the mixture was stirred at room temperature overnight. The resulting mixture was concentrated under reduced pressure to generate acid chloride (compound 3).

6-Bromohexanoic acyl chloride (compound 5) was stirred with BocNH(CH_2_)_2_NH_2_ (1.0 equiv) and K_2_CO_3_ (1.5 equiv) in CH_2_Cl_2_ at 0 °C for 30 min and 25 °C overnight. The sample was filtered to remove solids and washed with water, 1 M HCl, and brine. The residue was concentrated under reduced pressure to afford pale yellow crystals (compound 6).

A suspension of sodium hydride in oil (40% w/w, 3.5 equiv.) was suspended in dry THF, and a solution of 6 (1.0 equiv.) in dry THF was added dropwise at 0 °C over 30 min. The mixture was then stirred for an additional 12 h. The mixture was then cooled (ice), acidified (4 M HCl), and extracted with DCM. The combined organic extracts were washed with brine, dried, concentrated *in vacuo*, and purified by column chromatography to yield compound 7.

TFA (1.5 ml) was added to a solution of compound 7 (2.0 mmol, 1.0 equiv) in 5 ml of DCM. The mixture was stirred at room temperature for 2 to 3 h. A reaction was triggered by adding TLC. The reaction mixture was quenched by the addition of 2 M NaOH and the solution was extracted with ethyl acetate. The combined organic layers were washed with salt buffer, dried with Na_2_SO_4_, filtered, and concentrated under reduced pressure to obtain a residue. The residue was purified by column chromatography in ethyl acetate/petroleum to yield desired compound 8.

Desired compound 8 (1.05 equiv.) and triethylamine (2.0 equiv.) were combined in dichloromethane under nitrogen, resulting in a colorless suspension. Benzoyl chloride (1.0 equiv.) in dichloromethane was added dropwise over 10 min, and the solution was stirred overnight. The reaction mixture was quenched by the addition of water, and the mixture was extracted with CH_2_Cl_2_. The combined organic layers were dried over Na_2_SO_4_, filtered, and concentrated under reduced pressure to yield a residue. The residue was purified by column chromatography in ethyl acetate/petroleum to yield the desired compound 9.

### Nuclear magnetic resonance

A total of 10 mg of each compound was dissolved in 0.5 ml of CDCl_3_, and 1H NMR spectra were obtained on an AMX 400 NMR spectrometer from Bruker, Germany. ^1^H NMR data were reported as follows: chemical shift, multiplicity (s = singlet, d = doublet, t = triplet, q = quadruplet, m = multiplet), coupling constant (J values) in Hz, and integration. Chemical shifts (à) were reported with respect to the corresponding solvent residual peak at 7.26 ppm for CDCl_3_ for ^1^H NMR.

### Cytotoxicity assay

HepG2 and 293T cells were obtained from ATCC and cultured in Dulbecco’s modified Eagle’s medium supplemented with 10% fetal bovine serum and 1% penicillin-streptomycin (Gibco). Cells were maintained at 37 °C in a humidified atmosphere containing 5% CO2. Cells were seeded at a density of 5 × 10^3^ cells per well in 96-well plates and allowed to adhere for 24 h before treatment. Cells were treated with the test compounds at concentrations ranging from 1.5 to 400 μM for 48 h. Control wells were treated with an equivalent concentration of DMSO (vehicle control). Cell viability was assessed using the Cell Counting Kit-8. After 48 h of treatment, 10 μl of Cell Counting Kit-8 solution was added to each well, and the plates were incubated for 2 h at 37 °C. The absorbance was measured at 450 nm using a microplate reader. Cell viability was expressed as a percentage of the control group, and IC50 values were calculated using nonlinear regression analysis (GraphPad Prism 8.0). Data are presented as mean ± SD from three independent experiments, each performed in triplicate.

## Data availability

The data that support the findings of this study are available from the corresponding author upon reasonable request.

## Supporting information

This article contains supporting information.

## Conflict of interest

The authors declare that they have no conflicts of interests with the contents of this article.

## Supporting information

Table S1 and Figure S1-S8

ITC Raw Data
