# Supplementary material for: Substrate and inhibitor specificity of Plasmodium nucleoside transporters ENT1 orthologs
Source: J Biol Chem. 2024 Dec 24;301(2):108115. doi: 10.1016/j.jbc.2024.108115 (PMC11787452; doi:10.1016/j.jbc.2024.108115)
Supplement: Table S1 and Figure S1-S8 [file mmc1.docx]

**Supplementary Table 1.** **ITC binding** **data statistics. ND, undetectable.**

**
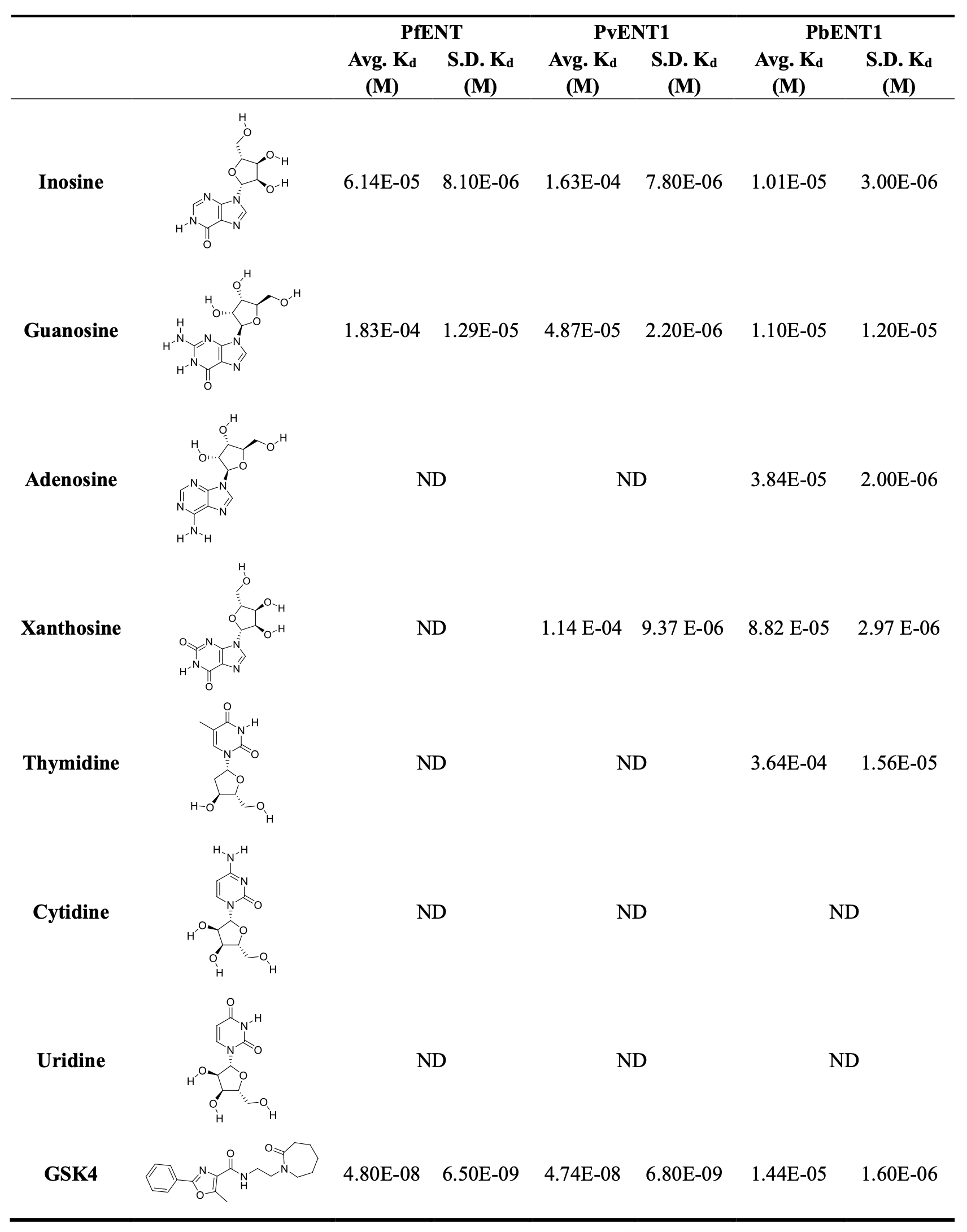
**

**
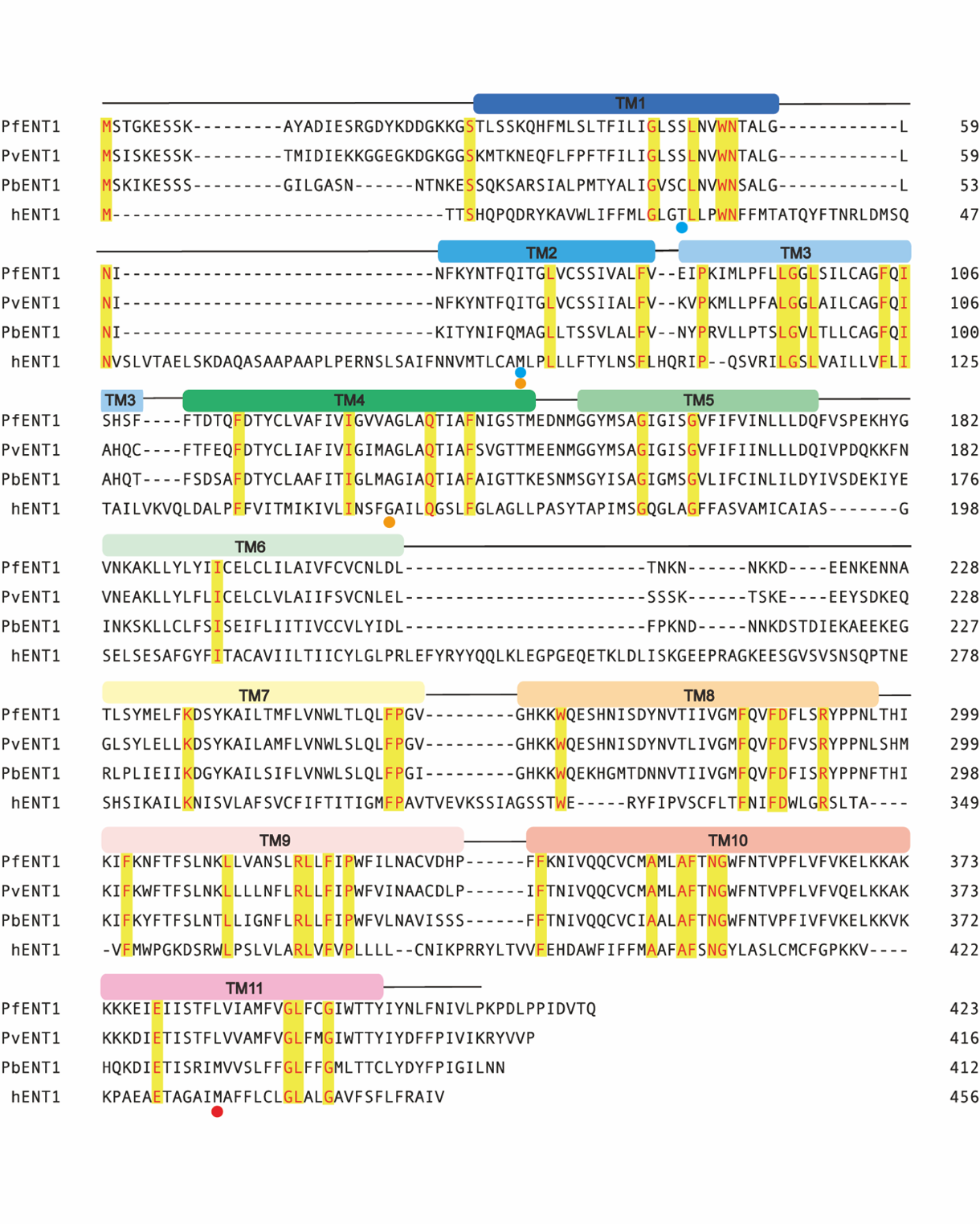
**

**Supplementary Fig. 1. Sequence alignment of ENTs.** PfENT1 (UniProt no. Q9NIH9), PvENT1 (UniProt no. A0A564ZY70), PbENT1 (UniProt no. A0A113SRM1), and hENT1 (UniProt no. Q99808). PfENT1 shares sequence identities of 75% and 60% with PvENT1 and PbENT1, respectively.


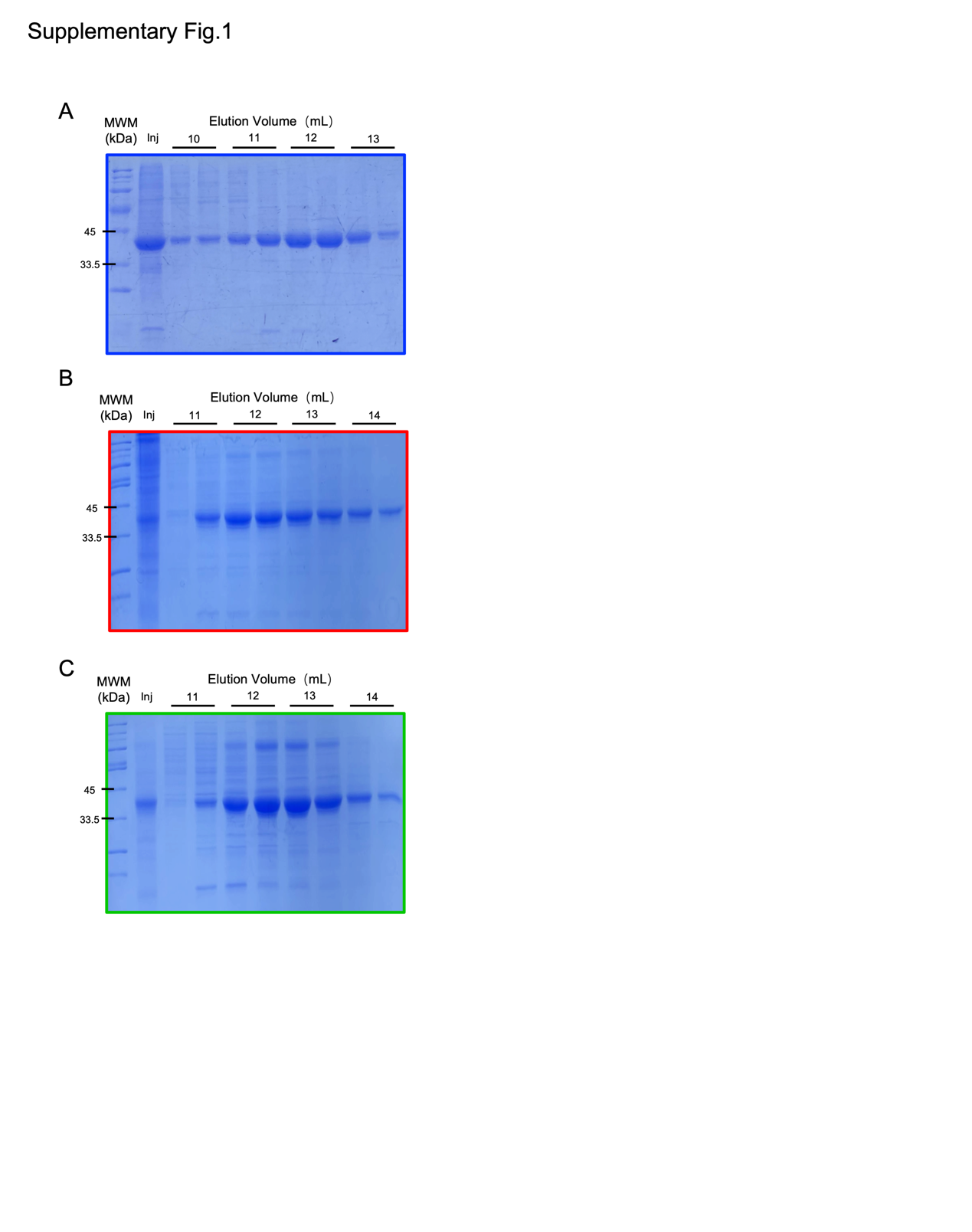


**Supplementary Fig. 2. Characterization of wild-type ENT1s. A.** Purification of wild-type PfENT1, wild-type PvENT1 (**B**) and wild-type PbENT1 (**C**) via size-exclusion chromatography (Superdex 200 10/300 Increase). The ENTs were purified in buffer containing 25 mM MES (pH 6.0), 150 mM NaCl, and 0.05% DDM. The peak fractions were subjected to SDS‒PAGE.


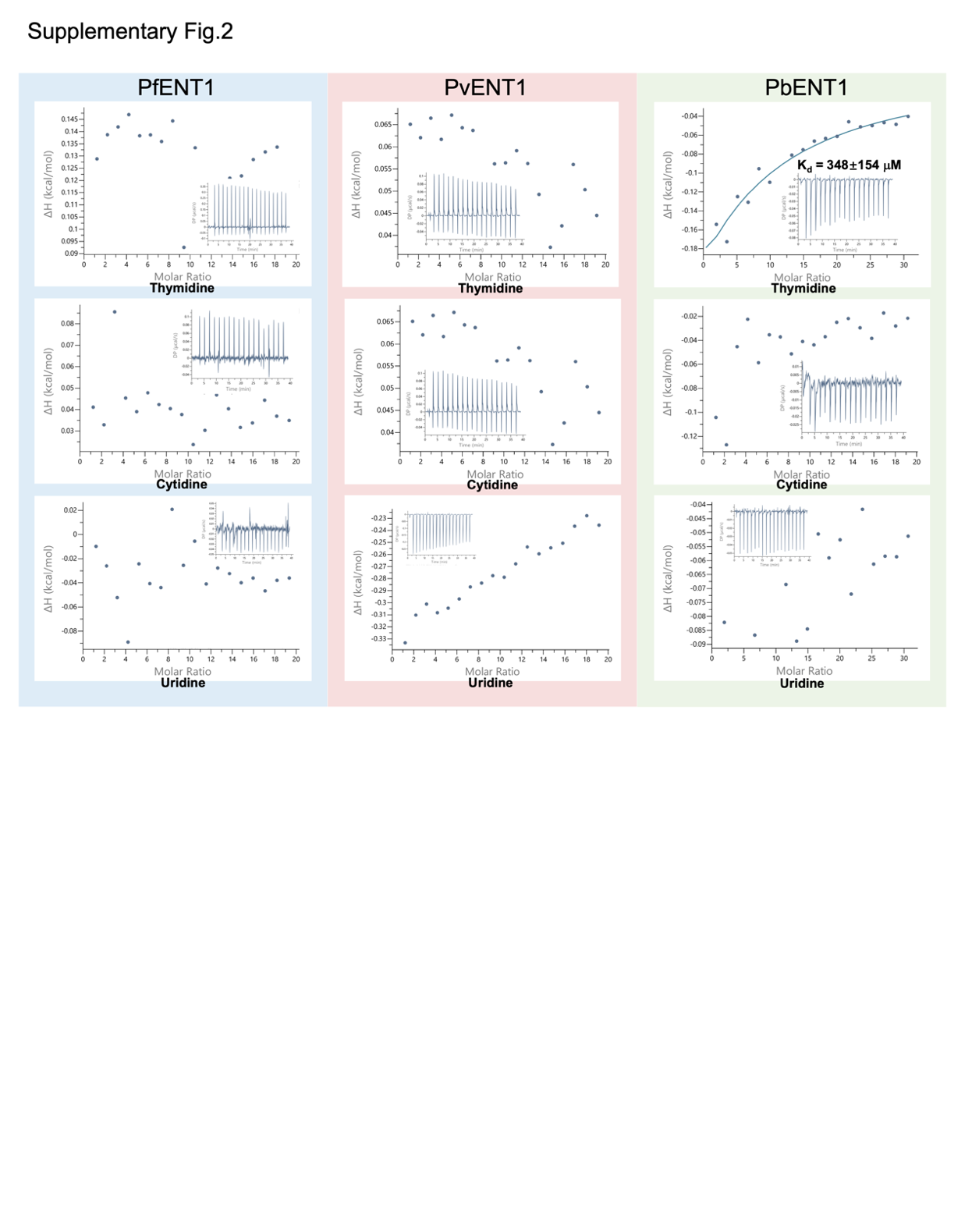


**Supplementary Fig. 3.** **ITC binding data and analyses.** Differential power traces and heats of injection traces of the specified nucleoside in the syringe of the ITC instrument. The error bars were obtained from a fit of the data points of the particular ITC experiments. The experimental conditions are described in the Methods section.


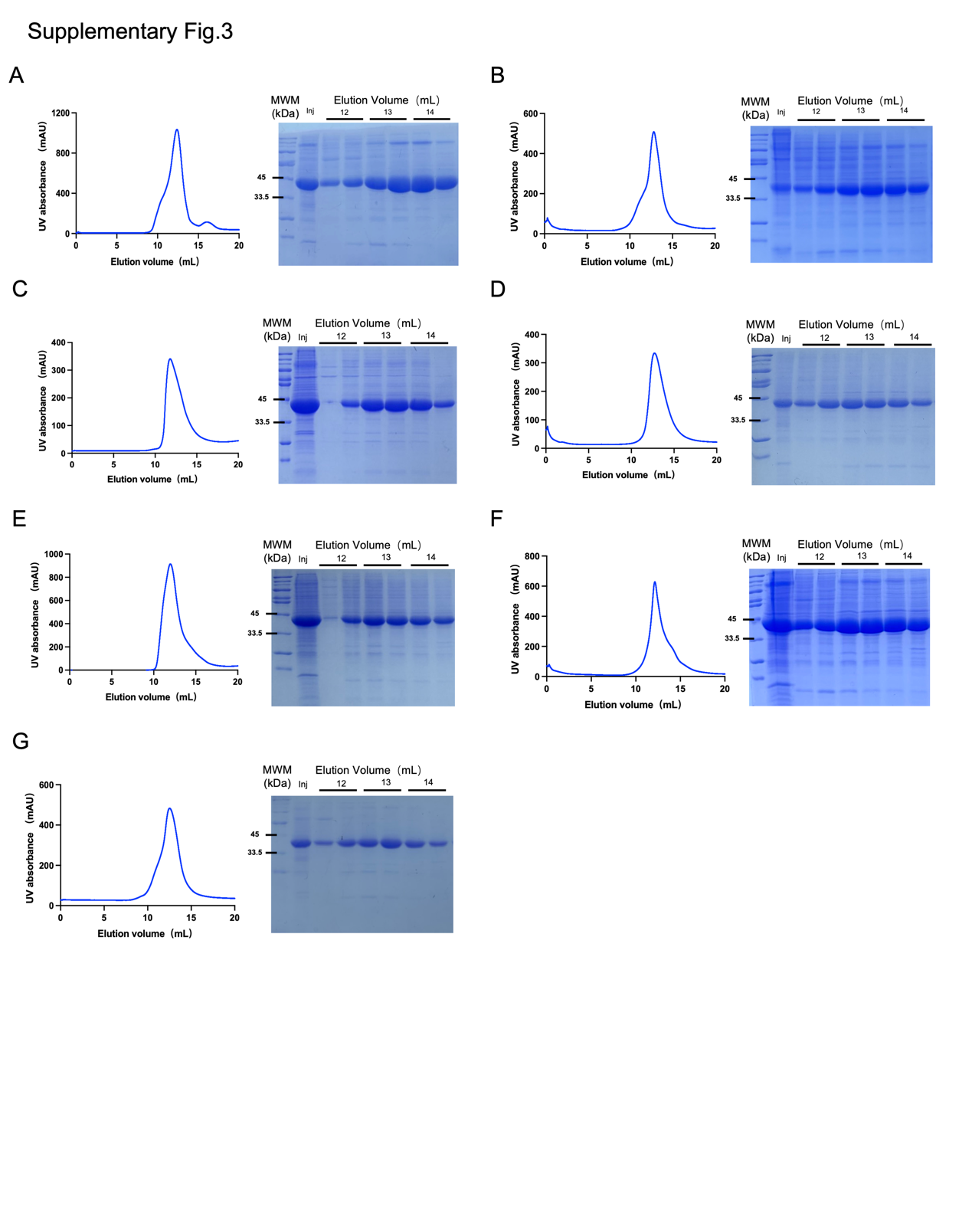


**Supplementary Fig. 4. Characterization of** **PfENT1_I70M_, PbENT1_M64I_, PfENT1****_S49C_, PbENT1_C43S_, PfENT1_I70M+A131G_, PfENT1_M389L_ and PbENT1_L388M._ A**. Purification of PfENT1_I70M_, PbENT1_M64I_ (**B**), PfENT1_S49C_ (**C**), PbENT1_C43S_ (**D**), PfENT1_I70M+A131G_ (**E**), PfENT1_M389L_ (**F**) and PbENT1_L388M_ (**G**) in size-exclusion chromatography (Superdex 200 10/300 Increase). PfENT1_I70M_ was purified in buffer containing 25 mM MES (pH 6.0), 150 mM NaCl, and 0.05% DDM. The peak fractions were subjected to SDS‒PAGE.


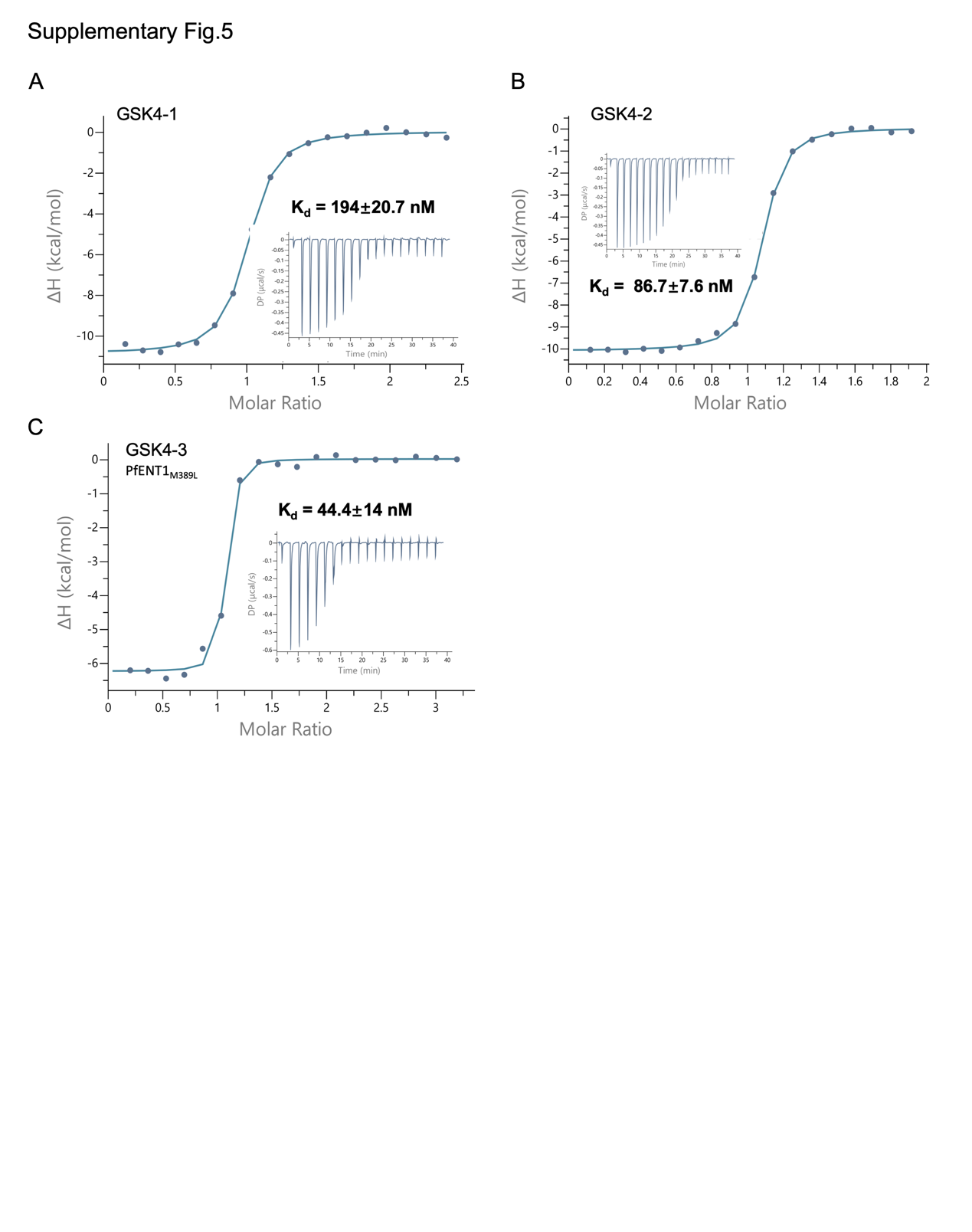


**Supplementary Fig. 5. ITC binding data and analyses for GSK4 anologs.** **A**. Binding of PfENT1 to GSK4-1 measured by ITC. **B**. Binding of PfENT1 to GSK4-2 measured by ITC. **C**. Binding of PfENT1_M389L_ to GSK4-3 measured by ITC.

**
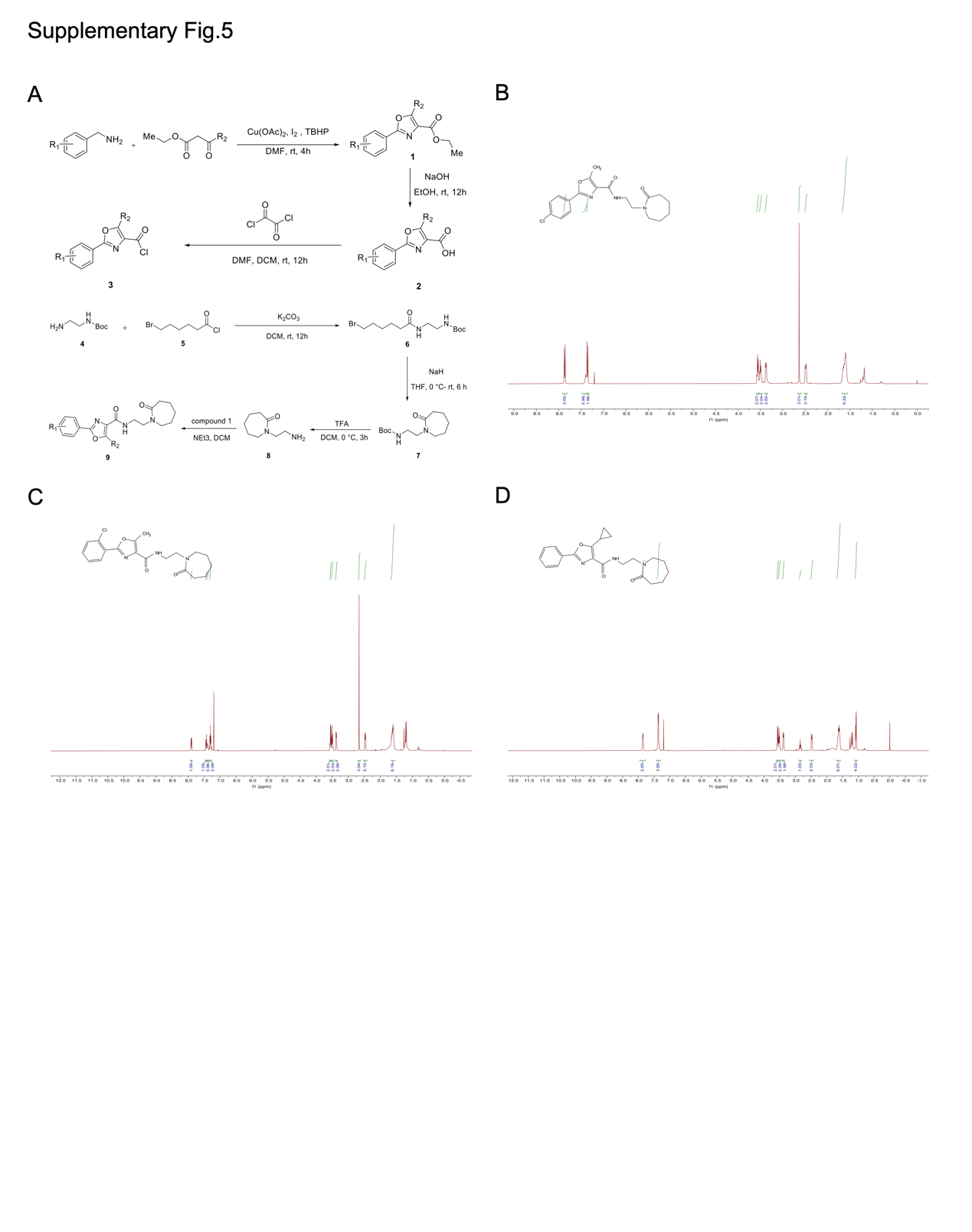
**

**Supplementary Fig. 6. Synthesis of GSK4-1, GSK4-2 and GSK4-3. A.** Nine-step chemical synthesis of GSK4-1, GSK4-2 and GSK4-3. **B.** NMR spectroscopy of GSK4-1. **C.** NMR spectroscopy of GSK4-2. **D.** NMR spectroscopy of GSK4-3.

**
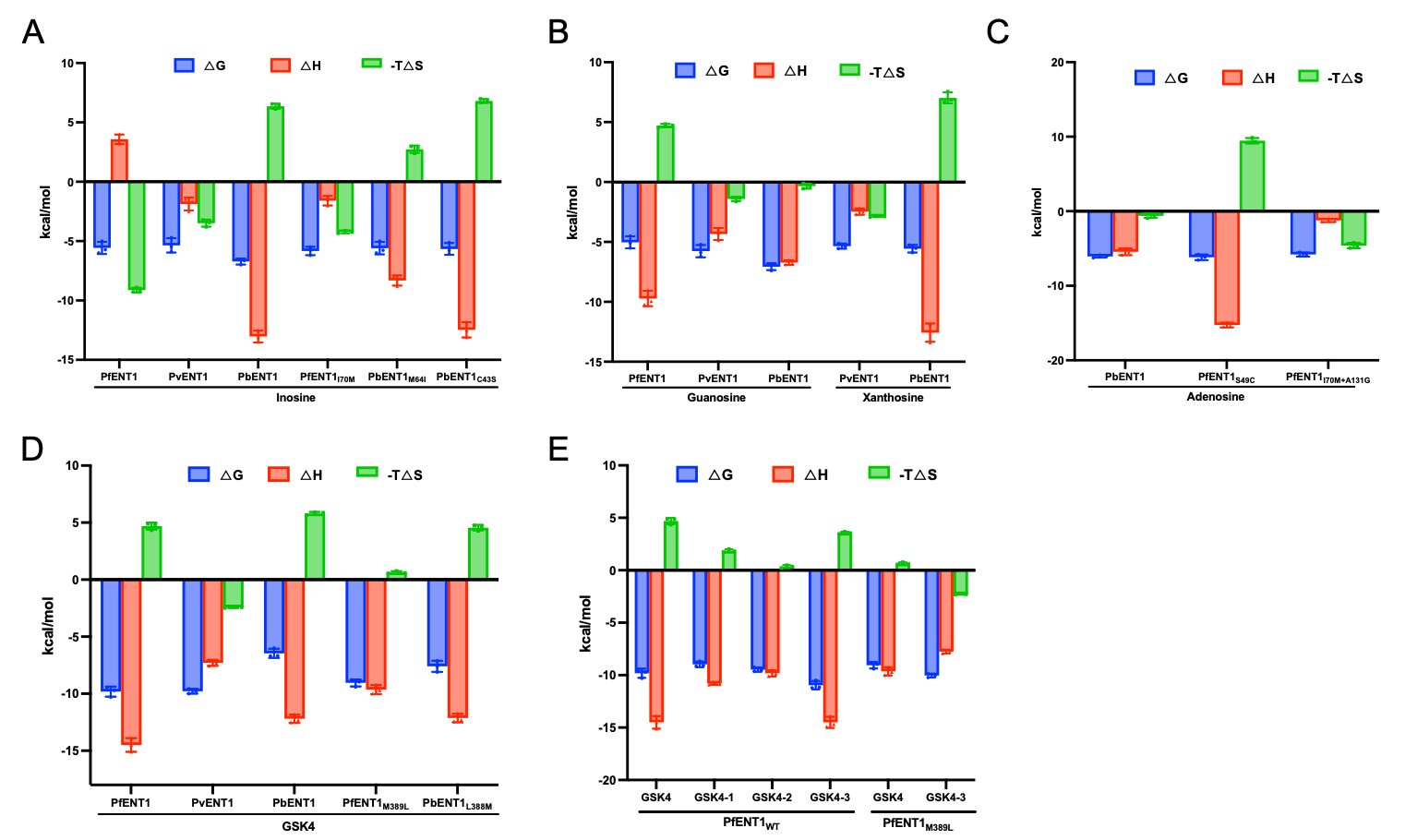
**

**Supplementary Fig. 7. Statistical diagram of thermodynamic parameters.** **A.** Thermodynamic parameters of inosine binding with ENT1s. **B.** Thermodynamic parameters of guanosine and xanthosine binding with ENT1s. **C.** Thermodynamic parameters of adenosine binding with ENT1s. **D.** Thermodynamic parameters of GSK4 binding with ENT1s. **E.** Thermodynamic parameters of inhibitors binding with PfENT1.

**
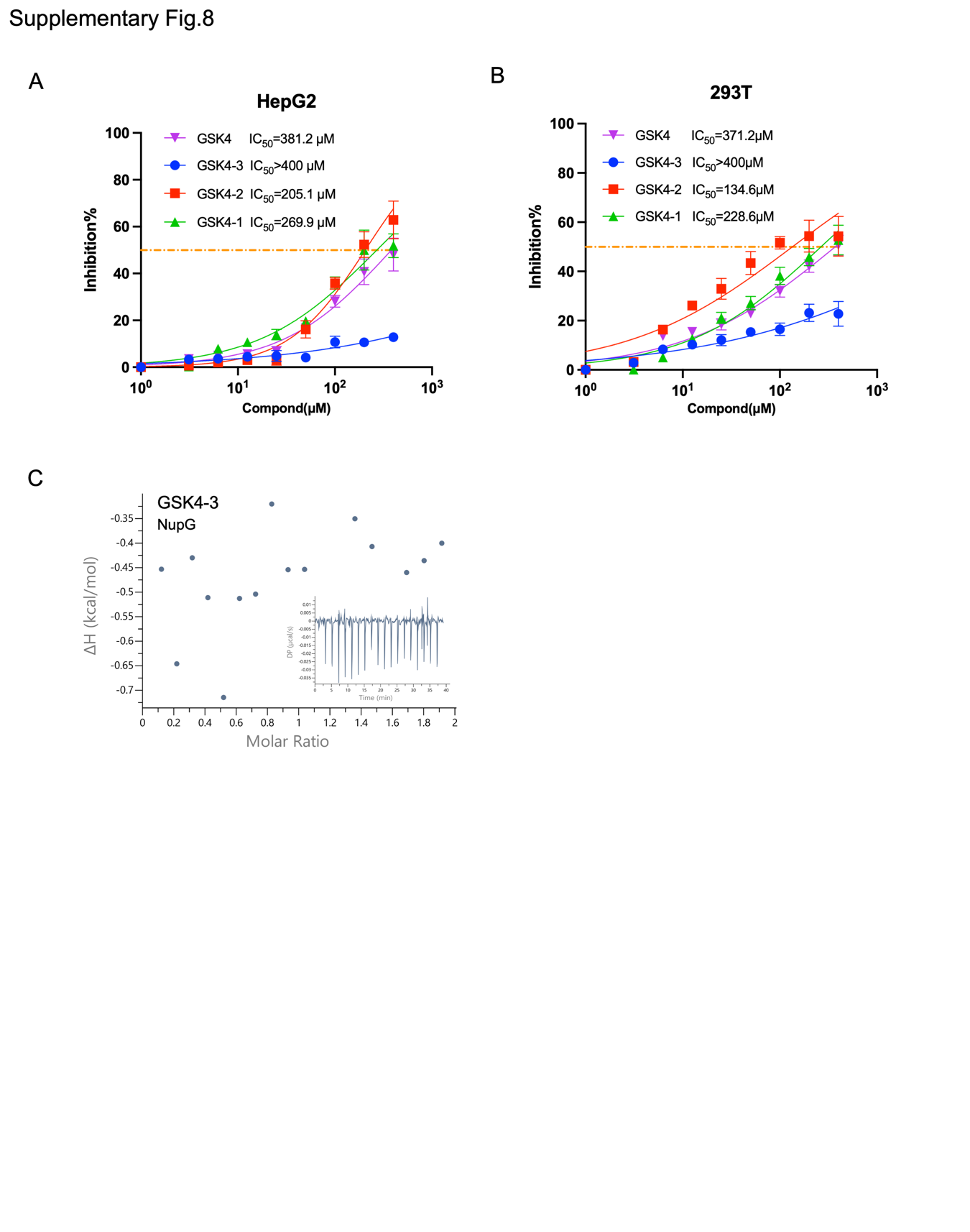
**

**Supplementary Fig. 8. Cytotoxicity and specificity evaluation of inhibitors. A.** Cytotoxicity assay of GSK4, GSK4-1, GSK4-2 and GSK4-3 on HepG2 cells. **B.** Cytotoxicity assay of GSK4, GSK4-1, GSK4-2 and GSK4-3 on 293T cells. **C.** GSK4-3 binding of NupG measured by ITC.
