# Supplementary material for: Substrate and inhibitor specificity of Plasmodium nucleoside transporters ENT1 orthologs
Source: J Biol Chem. 2024 Dec 24;301(2):108115. doi: 10.1016/j.jbc.2024.108115 (PMC11787452; doi:10.1016/j.jbc.2024.108115)
Supplement: ITC Raw Data [file mmc2.pdf]

Substrate and inhibitor specificity of *Plasmodium* nucleoside transporters ENT1 orthologues

ITC-Raw data

- The blue box represents the key graph depicted in the main text, and the K_d value provided is expressed as the mean ± standard deviation (SD) of three independent measurements.

Inosine

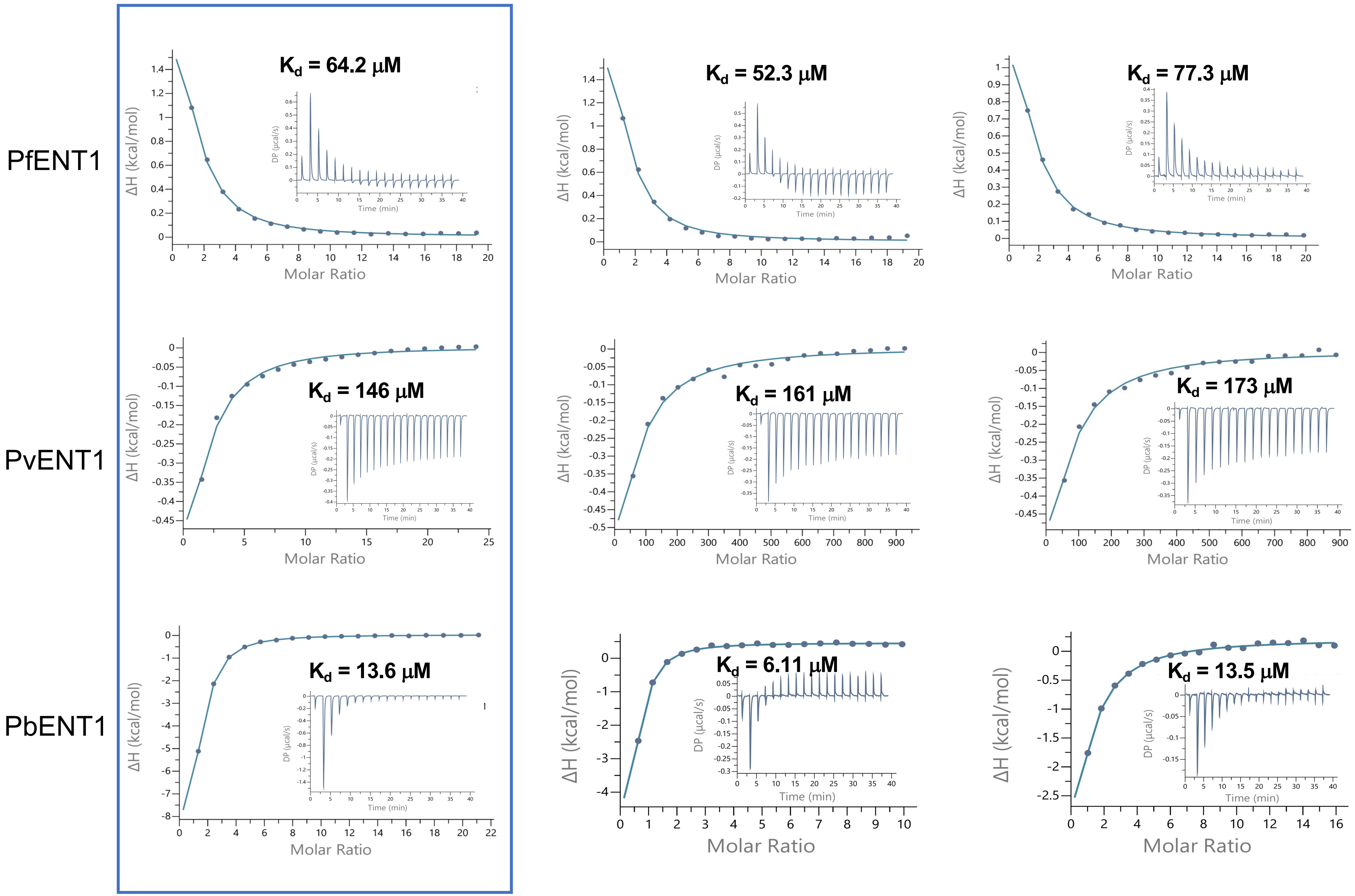

PfENT1
PvENT1
PbENT1
K_d = 64.2 μM
K_d = 52.3 μM
K_d = 77.3 μM
K_d = 146 μM
K_d = 161 μM
K_d = 173 μM
K_d = 13.6 μM
K_d = 6.11 μM
K_d = 13.5 μM
ΔH (kcal/mol)
Molar Ratio
DP (μcal/s)
Time (min)

Guanosine

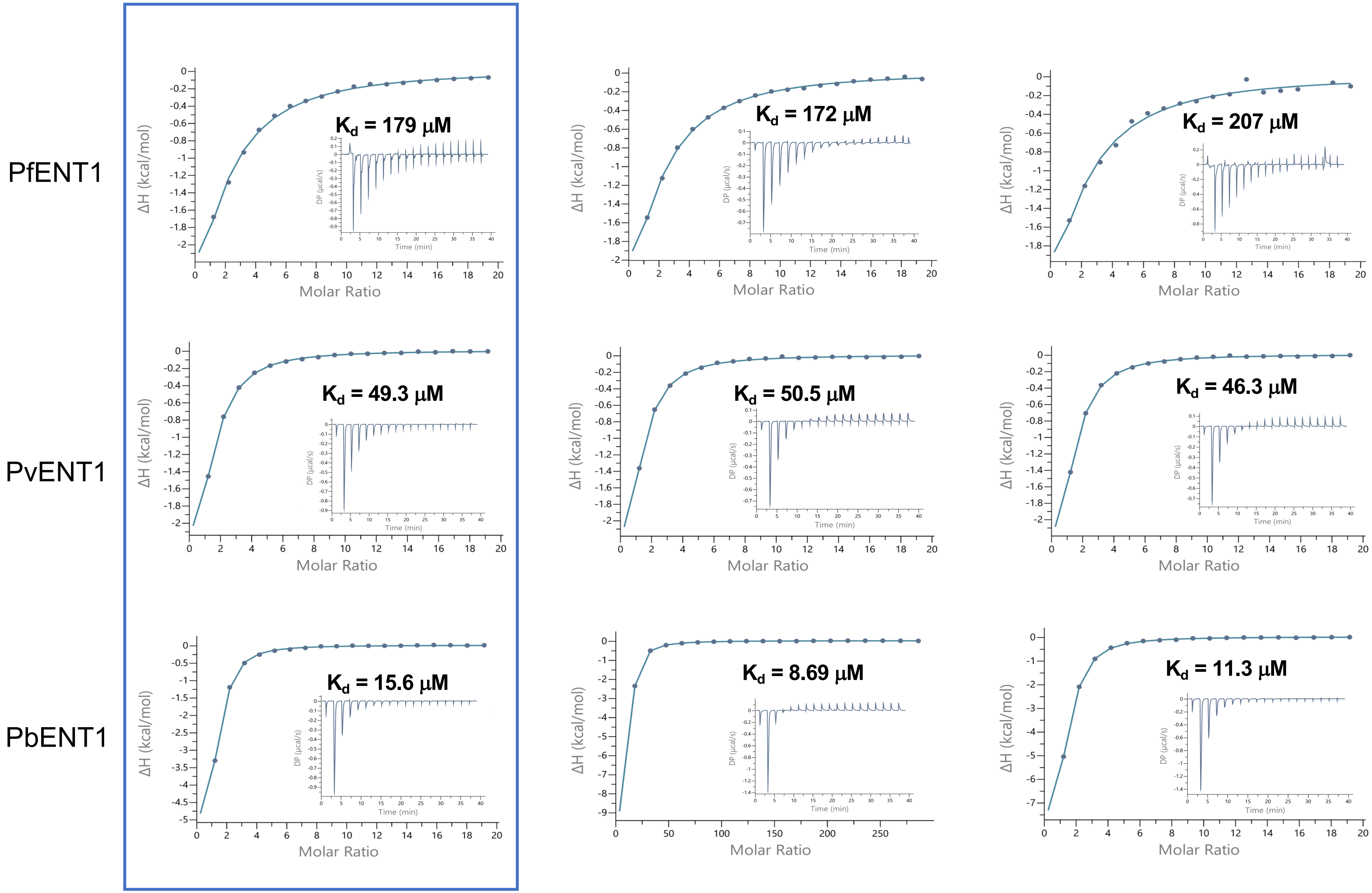
PfENT1
PvENT1
PbENT1
K_d = 179 μM
K_d = 172 μM
K_d = 207 μM
K_d = 49.3 μM
K_d = 50.5 μM
K_d = 46.3 μM
K_d = 15.6 μM
K_d = 8.69 μM
K_d = 11.3 μM
ΔH (kcal/mol)
Molar Ratio
DP (μcal/s)
Time (min)

Adenosine

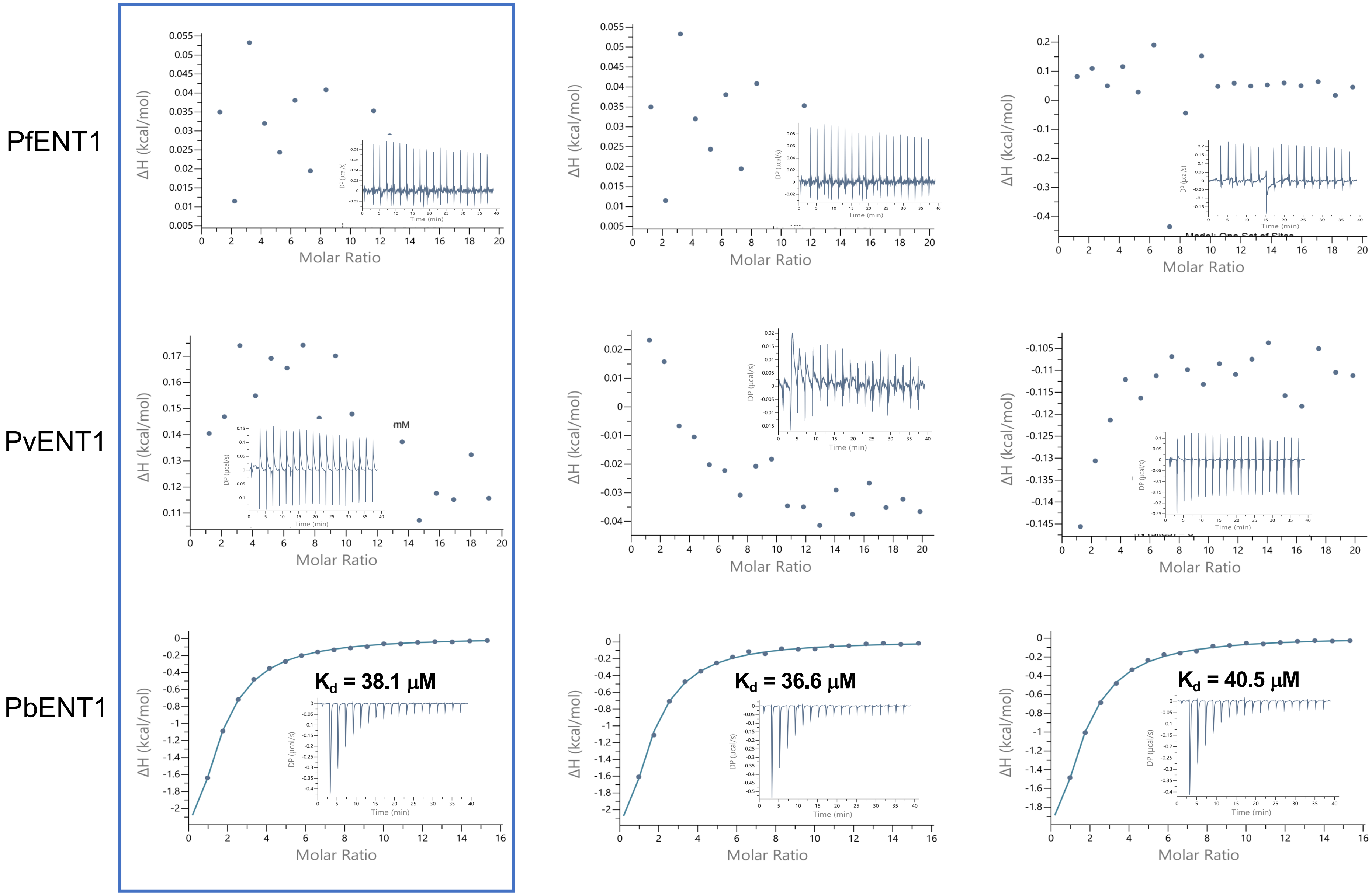
PfENT1
PvENT1
PbENT1
ΔH (kcal/mol)
Molar Ratio
DP (µcal/s)
Time (min)
mM
K_d = 38.1 µM
K_d = 36.6 µM
K_d = 40.5 µM

Xanthosine

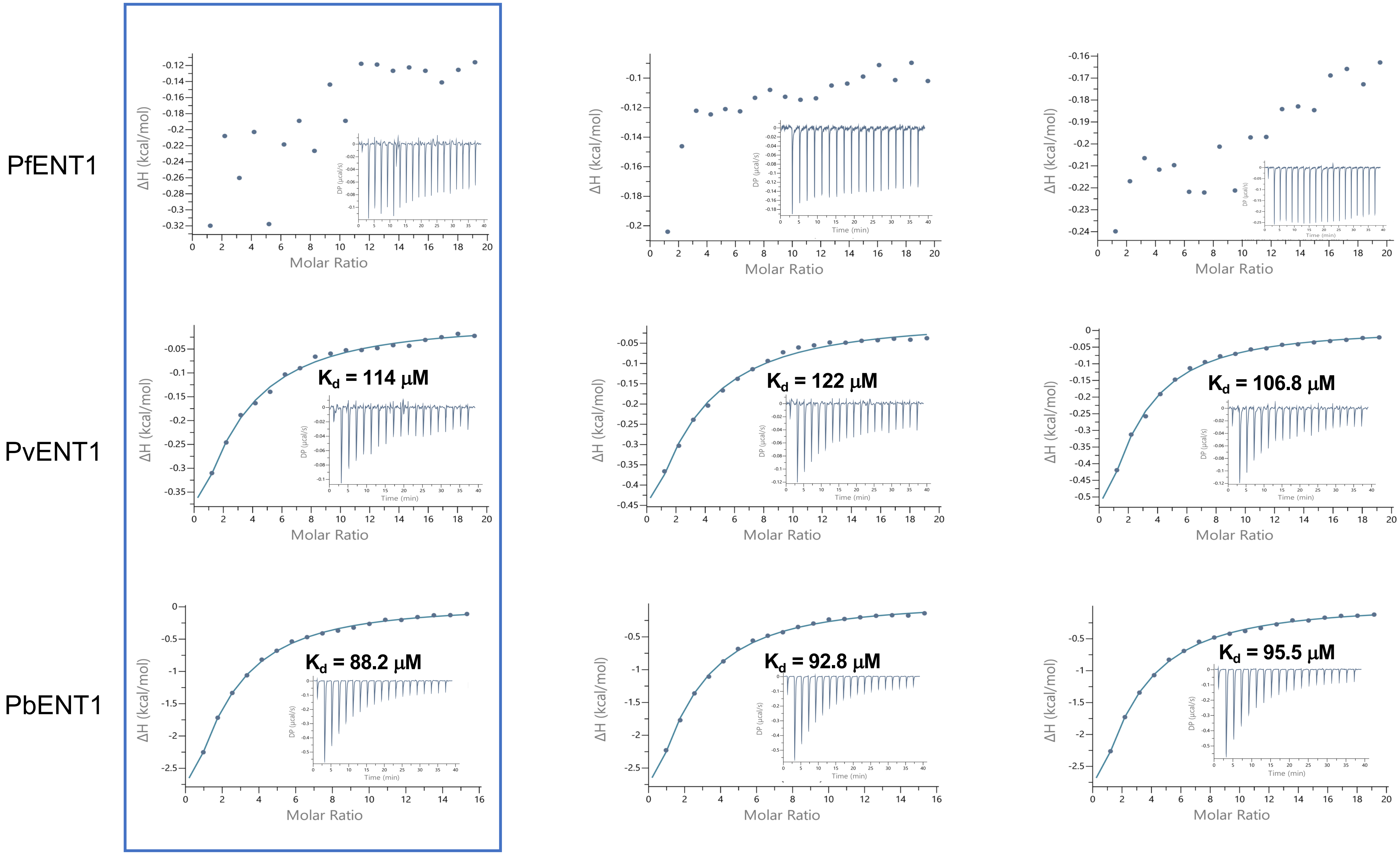

PfENT1
PvENT1
PbENT1
ΔH (kcal/mol)
Molar Ratio
DP (µcal/s)
Time (min)
K$_d$ = 114 µM
K$_d$ = 122 µM
K$_d$ = 106.8 µM
K$_d$ = 88.2 µM
K$_d$ = 92.8 µM
K$_d$ = 95.5 µM

GSK4

PfENT1

PvENT1

PbENT1

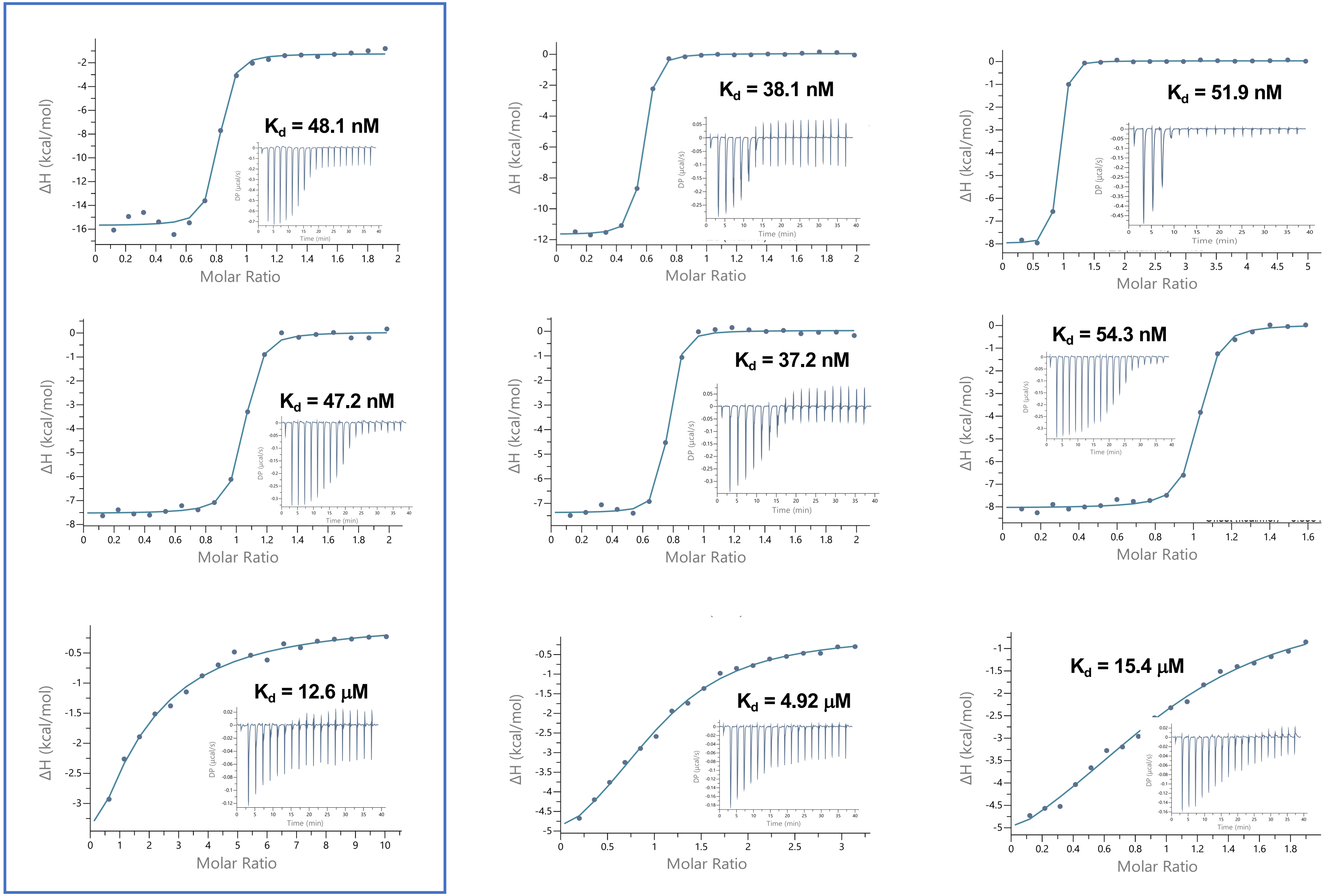

Inoisne

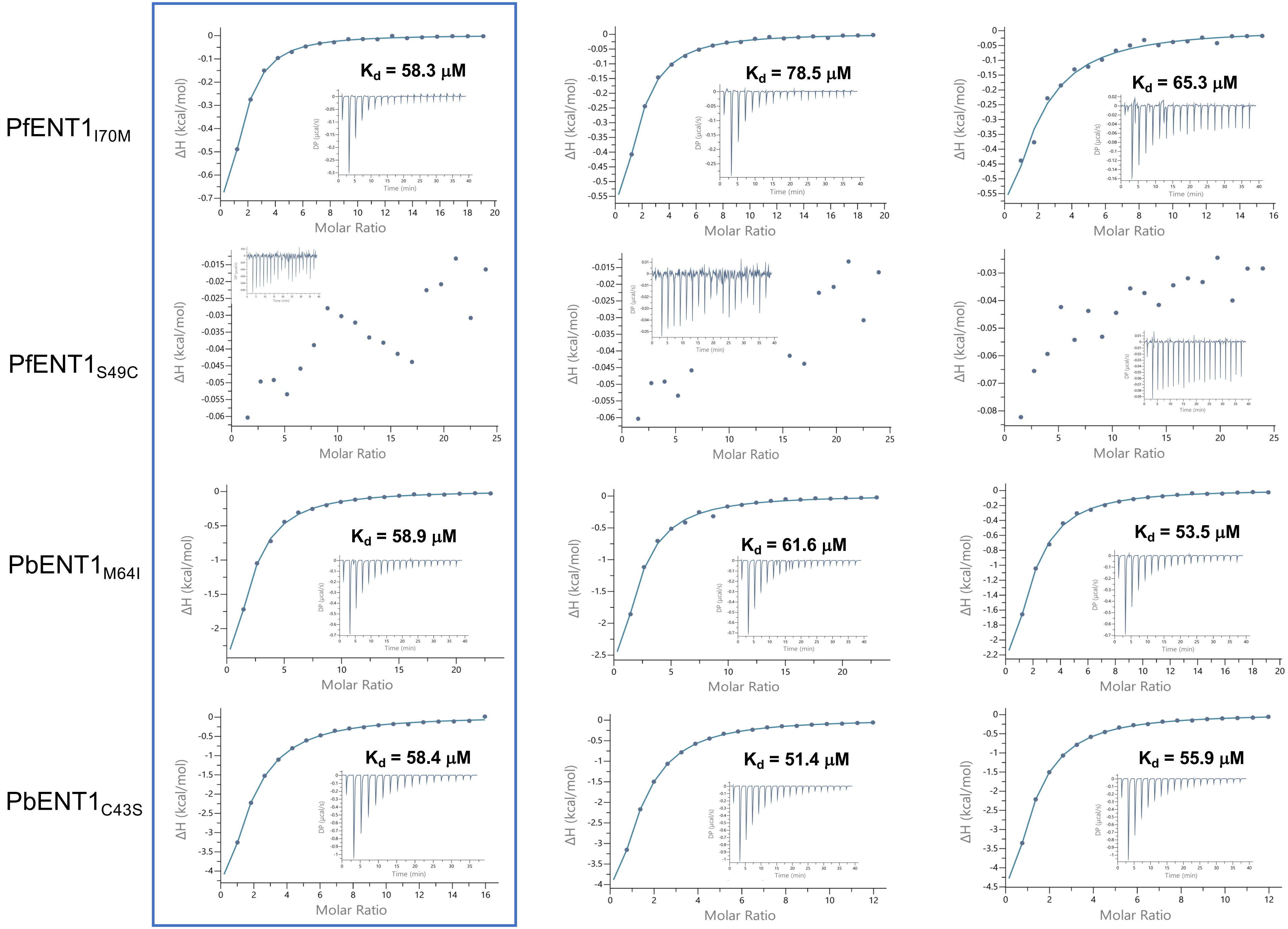

PfENT1$_{I70M}$
PfENT1$_{S49C}$
PbENT1$_{M64I}$
PbENT1$_{C43S}$
K_d = 58.3 μM
K_d = 78.5 μM
K_d = 65.3 μM
K_d = 58.9 μM
K_d = 61.6 μM
K_d = 53.5 μM
K_d = 58.4 μM
K_d = 51.4 μM
K_d = 55.9 μM
ΔH (kcal/mol)
Molar Ratio
DP (μcal/s)
Time (min)

Adenosine

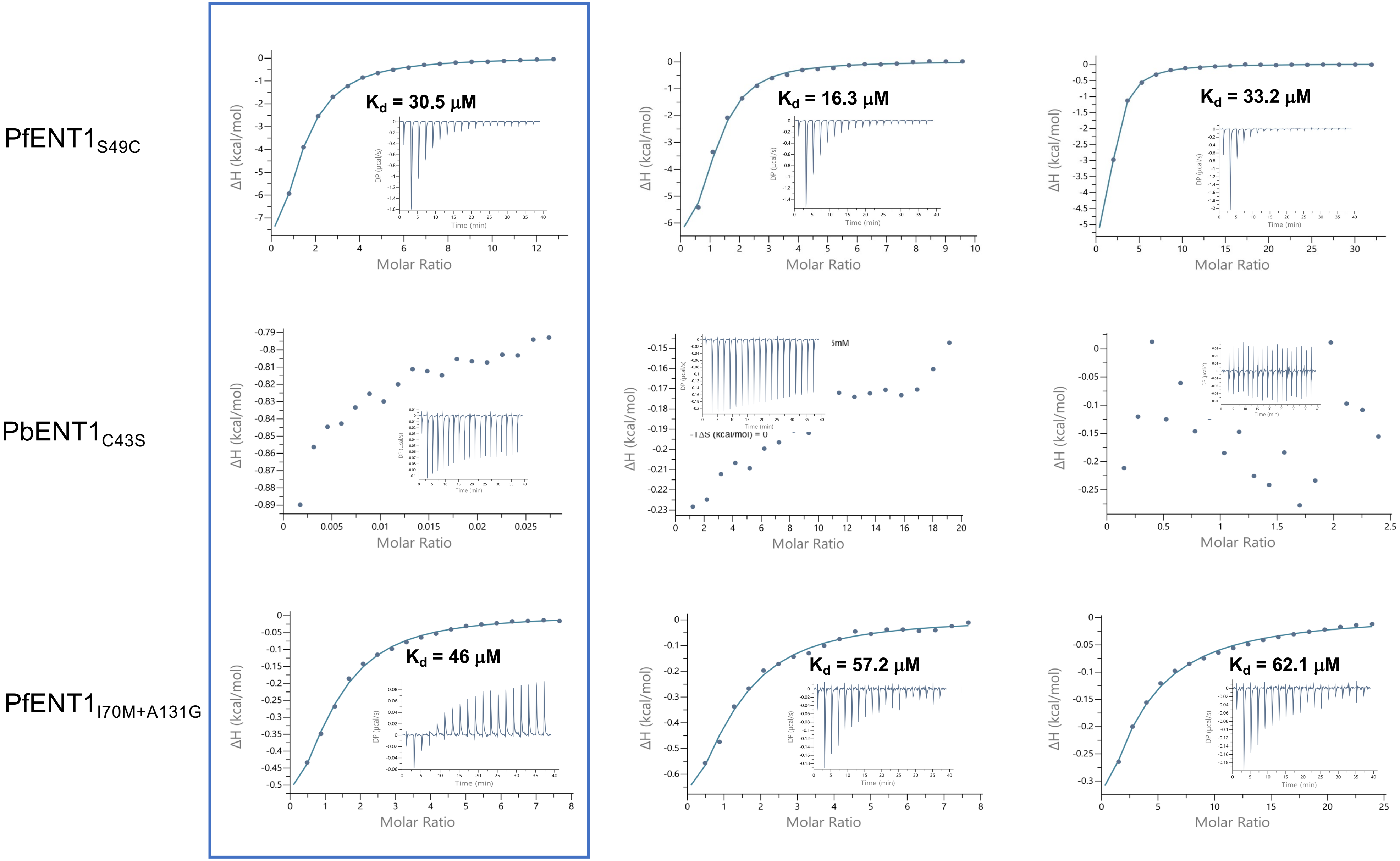
PfENT1S49C
PbENT1C43S
PfENT1I70M+A131G
Kd = 30.5 μM
Kd = 16.3 μM
Kd = 33.2 μM
Kd = 46 μM
Kd = 57.2 μM
Kd = 62.1 μM
ΔH (kcal/mol)
Molar Ratio
DP (μcal/s)
Time (min)

GSK4

$PfENT1_{M389L}$

$PbENT1_{L388M}$

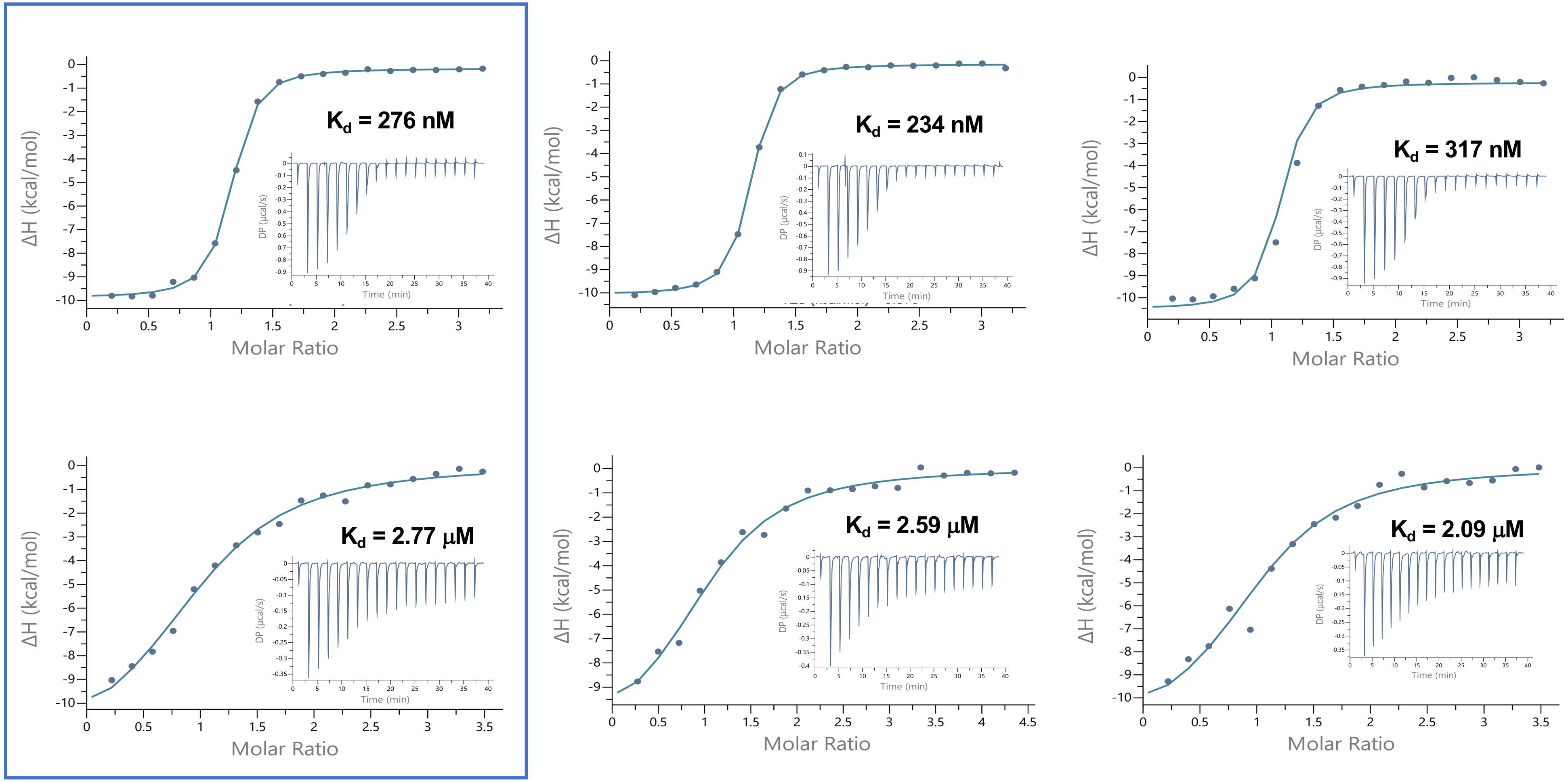

GSK4-3

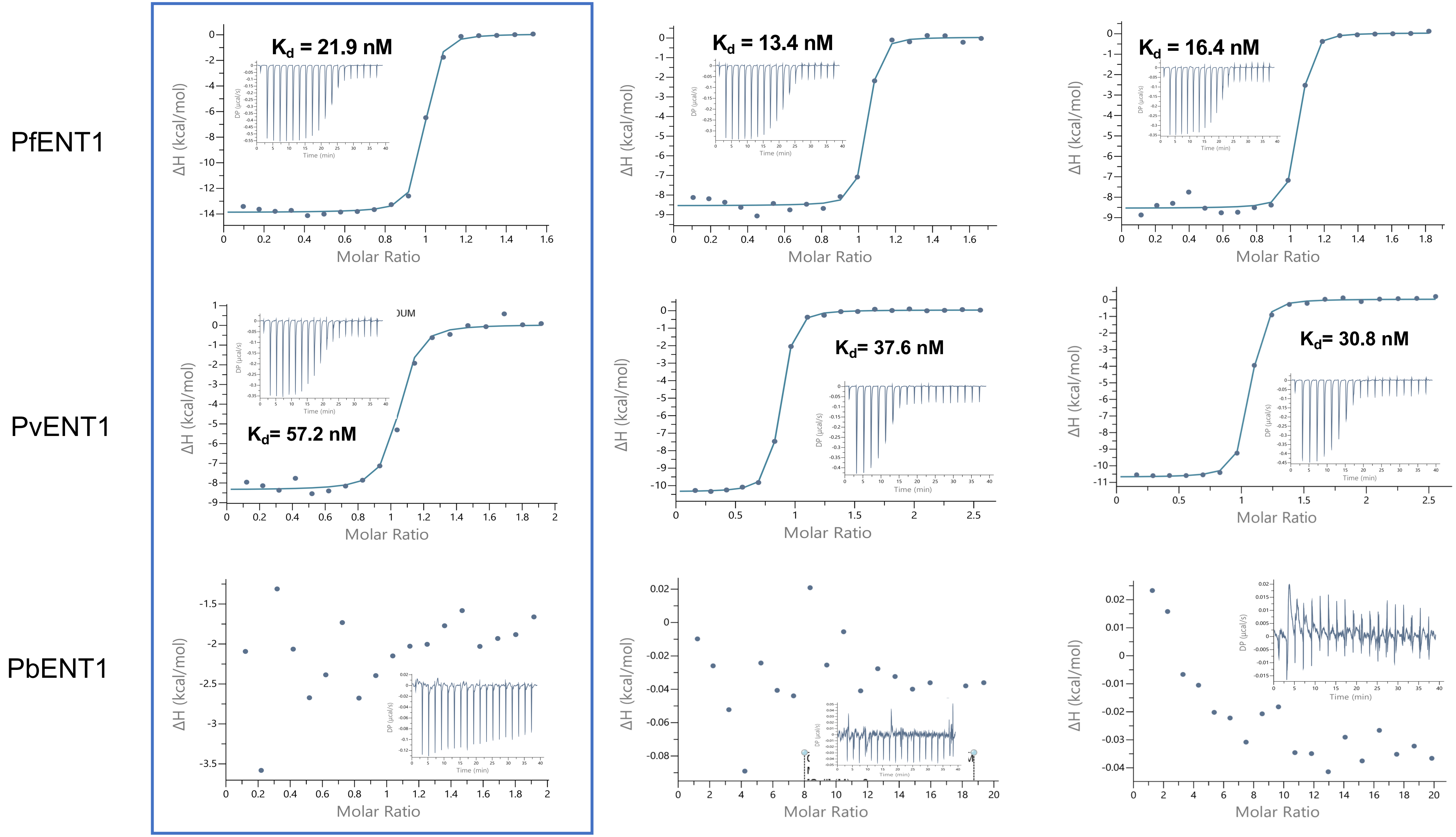
PfENT1
PvENT1
PbENT1
K_d = 21.9 nM
K_d = 13.4 nM
K_d = 16.4 nM
K_d= 57.2 nM
K_d= 37.6 nM
K_d= 30.8 nM
ΔH (kcal/mol)
Molar Ratio
DP (μcal/s)
Time (min)
